# Accuracy of congenital anomaly coding in live birth children recorded in European health care databases, a EUROlinkCAT study

**DOI:** 10.1007/s10654-023-00971-z

**Published:** 2023-02-18

**Authors:** Marian K. Bakker, Maria Loane, Ester Garne, Elisa Ballardini, Clara Cavero-Carbonell, Laura García, Mika Gissler, Joanne Given, Anna Heino, Anna Jamry-Dziurla, Sue Jordan, Stine Kjaer Urhoj, Anna Latos-Bieleńska, Elisabeth Limb, Renee Lutke, Amanda J. Neville, Anna Pierini, Michele Santoro, Ieuan Scanlon, Joachim Tan, Diana Wellesley, Hermien E. K. de Walle, Joan K. Morris

**Affiliations:** 1 Department of Genetics, University Medical Center Groningen, University of Groningen, Groningen, The Netherlands; 2 Institute for Nursing and Health Research, Ulster University, Northern Ireland, Newtownabbey, UK; 3 Department of Paediatrics and Adolescent Medicine, Lillebaelt Hospital, University Hospital of Southern Denmark, Kolding, Denmark; 4 Neonatal Intensive Care Unit, Paediatric Section, IMER Registry (Emilia Romagna Registry of Birth Defects), Department of Medical Sciences, University of Ferrara, Ferrara, Italy; 5 Rare Diseases Research Unit, Foundation for the Promotion of Health and Biomedical Research in the Valencian Region, Valencia, Spain; 6 Finnish Institute for Health and Welfare (THL), Helsinki, Finland; 7 Polish Registry of Congenital Malformations, Chair and Department of Medical Genetics, University of Medical Sciences, Poznan, Poland; 8 Faculty of Medicine, Health and Life Science, Swansea University, Swansea, UK; 9 Section of Epidemiology, Department of Public Health, University of Copenhagen, Copenhagen, Denmark; 10 Population Health Research Institute, St George’s University of London, London, UK; 11 IMER Registry, Centre for Epidemiology and Clinical Research, University of Ferrara and Azienda, Ospedaliero Universitario Di Ferrara, Ferrara, Italy; 12 Unit of Epidemiology of Rare Diseases and Congenital Anomalies, Institute of Clinical Physiology, National Research Council, Pisa, Italy; 13 University of Southampton and Wessex Clinical Genetics Service, Princess Anne Hospital, Southampton, SO16 5YA UK

**Keywords:** Accuracy, Coding, Congenital anomalies, Sensitivity, Positive predictive value

## Abstract

Electronic health care databases are increasingly being used to investigate the epidemiology of congenital anomalies (CAs) although there are concerns about their accuracy. The EUROlinkCAT project linked data from eleven EUROCAT registries to electronic hospital databases. The coding of CAs in electronic hospital databases was compared to the (gold standard) codes in the EUROCAT registries. For birth years 2010–2014 all linked live birth CA cases and all children identified in the hospital databases with a CA code were analysed. Registries calculated sensitivity and Positive Predictive Value (PPV) for 17 selected CAs. Pooled estimates for sensitivity and PPV were then calculated for each anomaly using random effects meta-analyses. Most registries linked more than 85% of their cases to hospital data. Gastroschisis, cleft lip with or without cleft palate and Down syndrome were recorded in hospital databases with high accuracy (sensitivity and PPV ≥ 85%). Hypoplastic left heart syndrome, spina bifida, Hirschsprung’s disease, omphalocele and cleft palate showed high sensitivity (≥ 85%), but low or heterogeneous PPV, indicating that hospital data was complete but may contain false positives. The remaining anomaly subgroups in our study, showed low or heterogeneous sensitivity and PPV, indicating that the information in the hospital database was incomplete and of variable validity. Electronic health care databases cannot replace CA registries, although they can be used as an additional ascertainment source for CA registries. CA registries are still the most appropriate data source to study the epidemiology of CAs.

**Supplementary Information:**

The online version contains supplementary material available at 10.1007/s10654-023-00971-z.

## Introduction

Congenital anomaly (CA) registries are set up with the specific aim of the monitoring and surveillance of CAs, to identify clusters of cases at the earliest possible opportunity, to evaluate health care policies and to identify possible risk factors for CAs. EUROCAT is a European network in which population-based CA registries collaborate in epidemiological surveillance and research. EUROCAT registries therefore collect very detailed data on CAs in live births, fetal deaths and termination of pregnancies for fetal anomalies, using multiple sources [1].

Electronic health care databases, such as hospital administrative data, are increasingly being used by researchers to investigate the epidemiology of CAs, for instance in studying space–time clusters [2] or geographic risk factors [3]. However, because these databases are not designed for research or surveillance, their data have often been found to be incomplete with respect to the coding of diagnoses such as CAs [4]. Recent studies in the USA and Australia estimated that over 90% of livebirths with a CA would be identified [5–7], but that the proportions identified with specific anomalies is much lower [8]. Andrade et al. [9] found only 37% of pregnancies affected with anencephaly were recorded in administrative claims and birth certificate data. Frohnert et al*.* [10] found that only 50% of children with atrial septal defects and 22% with of patent ductus arteriosus were identified in discharge data from a large urban medical center. A Canadian study reported slightly higher accuracy, but this was based on a restricted set of 16 CA groups and small study population [11]. In addition, Metcalfe et al*.* [12] showed that inpatient data (from hospitalizations) are adequate for ascertaining most, but not all CAs, while other sources of administrative data, particularly data from outpatient physician visits, were not adequate. Also, diagnosis codes in the hospital databases may be less precise which will result in many anomalies being categorized as ‘unspecified’ or ‘other’ [13]. A change from suspected diagnosis to confirmed diagnosis might not be reflected in the administrative data of the hospital and the inclusion of suspected or unconfirmed clinical diagnoses will over-estimate the prevalence of CAs.

Identifying which specific CAs can be accurately identified using only electronic health care databases will enable the surveillance of these anomalies to be performed worldwide, and not just in regions with CA registries. Similarly, identifying anomalies that are poorly reported in electronic health care databases (either under or over-reported) may limit their routine use or at least raise awareness of their limited accuracy.

In this study we evaluated the accuracy and the quality of the coding of CAs in hospital databases, compared to EUROCAT data, which were assumed to be the gold standard as registries use multiple ascertainment methods to identify cases. We estimated the overall and anomaly-specific accuracy for identifying CAs in hospital databases among children, born between January 2010 and December 2014 up to the first year of age.

## Methods

### Setting

This study was performed as part of the EUROlinkCAT project [14].

### Participating registries and hospital databases

Eleven EUROCAT registries in eight countries participated in this study: Emilia Romagna (Italy), Tuscany (Italy), Valencian Region (Spain), Finland, Poland, Wales (UK), Thames Valley (England, UK), Wessex (England, UK), East Midlands and South Yorkshire (EM&SY, England, UK), Northern Netherlands and Funen (Denmark). As part of the EUROlinkCAT project, these registries linked their CA data to regional or national electronic health care (hospital) databases [14]. Approvals for linkage were obtained locally by the registries. Poland was not able to perform the full linkage, but was able to link the diagnosis codes recorded in their CA registry with the diagnosis codes recorded in the National Health Fund.

A description of the eight hospital databases can be found in Table 1. The hospital databases in Italy and Valencian Region used International Classification of Diseases – Clinical Modification (ICD-9-CM) codes, and six used ICD-10 codes. The hospital databases in Denmark and Finland included inpatient and outpatient data and six included only inpatient data. Emilia Romagna, Valencian Region, Finland, Wales and Funen had access to the hospital data of the full reference population (i.e. all children in the region of coverage of the EUROCAT registry, including those not registered in the EUROCAT registry).

**Table 1:** Description of hospital databases that were linked to EUROCAT registries

Country/Region	Hospital database	Coverage	Hospital data		ICD coding in hospital data	EUROCAT Registry
			In-patient	Out-patient		
Italy	Scheda di Dimissione Osepdaliera	National with regional data control	Yes	–	ICD9-CM	Tuscany
						Emilia Romagna Registry of Birth Defects (IMER)
Spain	Conjunto Mínimo Básico de Datos (CMBD)	National but access to regional data	Yes	–	ICD9-CM	Valencian region
Finland	Terveydenhuollon hoitoilmoitusrekisteri	National	Yes	Yes	ICD10	Finland
Poland	Narodowy Fundusz Zdrowia	National	Yes	–	ICD10	Polish Registry Congenital Malformations (PRCM)
UK, Wales	Patient Episode Database for Wales (PEDW) (Inpatient data)	National (Wales)	Yes	–	ICD10	Congenital Anomaly Register and Information Service (CARIS)
UK, England	Hospital Episode Statistics, Admitted Patient Care	National (England)	Yes	–	ICD10	NCARDRS Thames Valley,
						NCARDRS Wessex,
						NCARDRS East Midlands & South Yorkshire,
Denmark	Landspatientregistret / Danish National Patient Register	National	Yes	Yes	ICD10	Funen
Netherlands	Landelijke basisregistratie ziekenhuiszorg (LBZ)	National	Yes	–	ICD10	Eurocat Northern Netherlands

^*^Polish registry of Congenital Malformation verifies the diagnosis from this database with the notifications from the doctors and from genetic counseling clinics. If there is no notification of the child the PRCM records the diagnosis in the database in accordance with strictly defined rules developed by PRCM. If the notification from the doctor is inserted in the future the verification will then be done

The linked data files were stored securely, either within the local registry or within the organization doing the linkage.

### Standardization

The EUROCAT registries code and classify their CA cases according to EUROCAT guidelines [15]. Using registry-specific STATA syntax scripts, local variables from each registry and hospital databases were standardized to a common data model based on the study protocol. Two registries, Finland and Wales, wrote their own standardization scripts based on the EUROlinkCAT template. Pre-specified tables with aggregated data were created through STATA scripts. The aggregated tables were transmitted to the Central Results Repository at the University of Ulster. All outputs were checked for consistency.

### Study population

Inclusion criteria for this study were all children, live born between 2010 and 2014, recorded in the EUROCAT registries and linked to hospital data and children identified in the hospital databases with any CA code, i.e. an ICD9-CM code in the range 740–759 or an ICD10 code from the Q-chapter, not recorded in EUROCAT registries. We restricted the diagnoses in all databases to those made in the first year of life.

We focused on seventeen specific anomalies, selected according to characteristics, which we expected to be related to the accuracy of CA coding in hospital databases:

1. anomalies detectable at birth (spina bifida, cleft lip with or without cleft palate, cleft palate, gastroschisis, omphalocele, clubfoot);
2. anomalies with a high prenatal detection rate (hypoplastic left heart syndrome (HLHS), unilateral renal agenesis, limb reduction defects)
3. anomalies usually diagnosed after discharge from the maternity unit (severe microcephaly, ventricular septal defect (VSD), Hirschsprung’s disease)
4. anomalies that present in variable form between normal and abnormal (atrial septal defect (ASD), congenital hydronephrosis, hypospadias)
5. chromosomal anomalies (Down syndrome)
6. mild anomalies (polydactyly).

For each of the seventeen specific anomalies the ICD9-CM or ICD10 diagnosis codes were defined (see supplementary file 1 (table A)).

### Outcomes

**Fig. 1:**
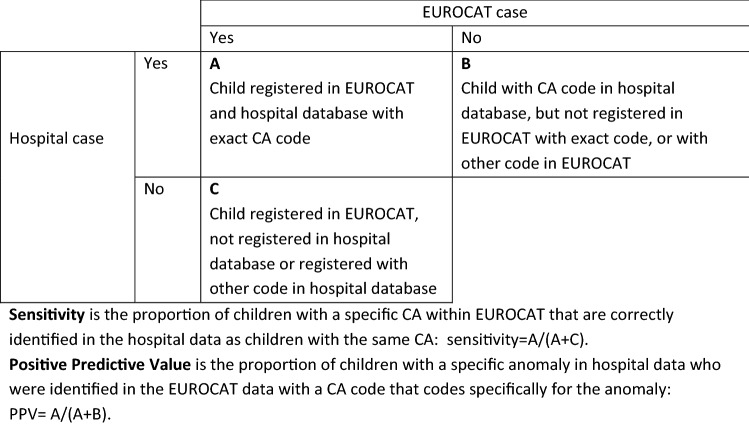
Comparison of congenital anomaly (CA) codes recorded in hospital databases to CA codes recorded in EUROCAT where the EUROCAT anomaly coding is considered as the gold standard

To assess the accuracy of the hospital data, we compared the codes in the hospital database to codes recorded in the CA registries data for the 17 specific CAs by calculating sensitivity and positive predictive value (PPV) for each specific anomaly, assuming the EUROCAT anomaly coding is the gold standard. Sensitivity is an estimate of completeness of the hospital data. It measures the proportion of children with a specific CA within EUROCAT that are correctly identified in the hospital data as children with the same CA (see Fig. 1). For the sensitivity analyses, only live born EUROCAT cases with isolated anomalies (i.e. not part of a chromosomal disorder or syndrome and no other unrelated anomalies present) were included. The PPV is the proportion of children with a specific anomaly in hospital data who were present in the EUROCAT data with a CA code that codes specifically for the anomaly (see Fig. 1). The PPV is an estimate of the quality or validity of CA coding in hospital databases. An accurate estimate of PPV can only be calculated using hospital data from the same reference population from which the EUROCAT cases were derived. Therefore PPV for each of the specific anomalies included was calculated for registries who had access to hospital data from the full reference population: Emilia Romagna, Valencian Region, Finland, Wales and Funen. Registries who had no access to the hospital data of children not registered in EUROCAT or only from a sample of the reference population were not included in the PPV analyses.

### Analysis

We used a stepwise approach to calculate an estimate of the accuracy of congenital anomaly coding in European hospital databases.

1. We first calculated sensitivity and PPV and 95% confidence interval (CI) per registry for each anomaly.
2. We then calculated pooled estimates for sensitivity and PPV for three groups of registries. The classification of the registries in three groups was based on whether the hospital database used ICD10 or ICD9 coding systems and whether the hospital database was a mainly independent source for the EUROCAT registry, or a direct source of ascertainment. These characteristics may be related to the accuracy of CA coding.
  - Group I consisted of the three registries where the hospital databases used ICD-9-CM coding: Tuscany, Emilia Romagna and Valencian Region.
  - Group II consisted of Finland and Poland, which both use the electronic hospital database as a direct and important source of ascertainment for their cases.
  - Group III consisted of the remaining registries: Thames Valley, EM&SY, Wessex, Wales, Funen and Northern Netherlands.
3. The pooled estimates of these three groups were then used to calculate an overall estimate. The overall pooled estimate is only presented if the *p*-value for heterogeneity between groups was ≥ 0.05. If the *p*-value for heterogeneity between groups was < 0.05, indicating significant differences between the three groups, only the group estimates for sensitivity and PPV are provided.

All random effects meta-analyses were performed using the “metaprop” package in STATA, version 15 with the Freeman-Tukey Double Arcsine Transformation to stabilize the variances and exact confidence intervals for the individual studies. The Northern Netherlands had to round all numbers to the nearest 5 because of release restrictions for small numbers. Therefore, we included Northern Netherlands data in the pooled analyses only for anomalies with ≥ 10 isolated cases. Results are reported for Wales, the three English registries and Funen, taking any restrictions on the release of aggregated data and analytic results for small numbers into account [16]. Based on the distribution of the sensitivity and PPV estimates of the specific anomalies per registry (first step in the analyses), we classified sensitivity and PPV estimates of ≥ 85% as high.

## Results

In Table 2 the results of the linkage to the hospital databases are presented for each registry. Eight registries linked more than 90% of their live born EUROCAT cases, with highest linkage achieved in Finland and Funen (> 99%). A CA code was recorded in the hospital data in the first year of life for 57% of the linked EUROCAT cases in Northern Netherlands and up to 96% in Valencian Region.

**Table 2:** Result of linkage of EUROCAT registries to hospital databases

Registry or region	All EUROCAT Livebirths	Linked to electronic hospital data		Linked cases: Congenital anomaly code in electronic hospital data	
	n	n	%	n	%
Tuscany, Italy	2,469	2248	91.0%	1,838	81.8%
Emilia Romagna, Italy	4,413	4,047	91.7%	3,663	90.5%
Valencian Region, Spain	4,303	4,205	97.7%	4,041	96.1%
Finland	12,752	12,654	99.2%	11,384	90.0%
Poland^a^	12,047	12,047	100%	9,636	80.0%
Wales, UK	3,451	2,684	77.8%	2,126	79.2%
Thames Valley, UK	1,497	1,419	94.8%	1,183	83.4%
Wessex, UK	2,030	1,878	92.5%	1,547	82.4%
East Midlands & South Yorkshire, UK	3,273	3,160	96.5%	2,497	79.0%
Funen, Denmark	479	476	99.4%	448	94.1%
Northern Netherlands^b^	585	505	86.3%	290	57.4%

Number (n) and % of EUROCAT livebirth cases linked to hospital data and number and % of linked EUROCAT livebirth cases with congenital anomaly code in hospital data, birth years 2010–2014

^a^Polish registry of congenital malformations receives data from many sources, including entities providing health services in the field of neonatology, obstetrics, clinical genetics, pediatric surgery, orthopedics, pediatrics, pediatric cardiology, ophthalmology, pediatric neurology, pediatric otolaryngology, intensive pediatric therapy, primary care, pathomorphology, lung diseases, endocrinology and pediatric diabetes, child and adolescent psychiatry and voivodship branches of the National Health Fund. In this study we compared the diagnosis registered in the PRCM with the codes registered in the NHF

^b^Northern Netherlands included birth years 2013–2014

### Sensitivity

Results of pooled estimates for sensitivity are presented in Table 3. The results per registry are presented in supplementary file 2 (figures B). Highest overall pooled sensitivity was found for Hirschsprung’s disease. A condition diagnosed after discharge from the maternity unit, but on average almost 100% of the EUROCAT children with Hirschsprung ‘s disease were registered in hospital databases with the exact ICD code. For eight of the twelve isolated anomalies where an overall pooled estimated could be calculated, the overall pooled sensitivity was over 85% (Hirschsprung’s disease, omphalocele, gastroschisis, cleft lip with or without cleft palate, HLHS, spina bifida, cleft palate and Down syndrome). For polydactyly and hypospadias the overall sensitivity was lower than 80%. Sensitivity was variable between the three groups for clubfoot, unilateral renal agenesis, limb reduction defects, severe microcephaly and hydronephrosis. In general, the group estimates for these anomalies were highest in group I *-ICD9 hospital codes* and II-*registries using data from healthcare databases*, and lowest in group III-*other*. For clubfoot the sensitivity was lowest in group I-*ICD9 hospital codes* and group III-*other*.

**Table 3:** Estimates for sensitivity for isolated congenital anomalies, diagnosed in the first year of life

Congenital anomaly	Pooled estimate for sensitivity							
	Group I – ICD9 registries		Group II – Data from health care databases		Group III – Other registries		Overall pooled estimate	
	%	(95% CI)	%	(95% CI)	%	(95% CI)	%	(95% CI)
Spina bifida	77	(50–97)	86	(79–92)	95^a^	(85–100)	89	(82–95)
Cleft lip with or without cleft palate	97	(93–100)	94	(92–96)	97	(95–98)	96	(94–98)
Cleft palate	72	(49–90)	91	(88–93)	97	(92–100)	90	(83–96)
Gastroschisis	–^b^		98	(94–100)	98^a^	(94–100)	98	(96–100)
Omphalocele	–^b^		93	(85–99)	100^a^	(95–100)	99	(94–100)
Clubfoot	38	(33–43)	89	(86–91)	39	(19–61)	Nc	
Hypoplastic left heart syndrome	90	(66–100)	92	(88–96)	90^a^	(80–97)	93	(88–97)
Unilateral renal agenesis	84	(75–91)	71	(65–77)	41^a^^,c^	(27–55)	Nc	
Limb reduction defects	80	(64–92)	57	(49–64)	40	(31–49)	Nc	
Severe microcephaly	89	(81–96)	72	(61–82)	78^a^	(54–96)	Nc	
VSD	88	(82–93)	84	(83–85)	71	(51–87)	78	(69–86)
Hirschsprung disease	100	(94–100)	98	(94–100)	100^a^	(96–100)	100	(98–100)
ASD	80	(63–93)	81	(78–83)	62	(39–83)	73	(63–81)
Congenital hydronephrosis	39	(19–62)	85	(82–87)	55	(39–70)	Nc	
Hypospadias	81	(65–92)	71	(68–74)	72	(44–93)	75	(62–86)
Down syndrome	91	(85–96)	86	(84–89)	91	(85–96)	91	(85–96)
Polydactyly	77	(70–84)	72	(69–75)	77	(68–86)	76	(71–81)

Group I: Tuscany, Emilia Romagna, Valencian Region

Group II: Finland, Poland

Group III: Wessex, East Midlands and South Yorkshire, Thames Valley Wales, Funen and Northern Netherlands

*CI* confidence interval, *VSD* ventricular septum defect, *ASD* atrial septum defect, *Nc* overall pooled estimate was not calculated because of heterogeneity between the group estimates

^a^NNL data excluded because of number of cases < 10

^b^In group I no group estimate was calculated, because the hospital database linked to ER and Tuscany used another code for abdominal wall defects. Overall pooled estimate is based on group II and III alone

^c^Funen data excluded

When sensitivity was low for a specific anomaly, cases were recorded in the hospital database with other, but not exact, CA codes or no CA code was recorded. Low overall sensitivity estimates for polydactyly and hypospadias could mainly be attributed to no recording of a CA code in the hospital data. Low sensitivity for clubfoot was mainly caused by the use of other, not exact clubfoot codes. For the other anomalies both non-recording and use of other codes attributed to low sensitivity estimates (data not shown).

### Positive predictive value

For the registries with access to hospital data for the full reference population, highest PPVs were frequently found in group II-*registries using data from health care databases*, see Table 4 and supplementary file 3 (figures C). Gastroschisis had the highest overall group and pooled estimates for PPV (100%, 95% CI: 98–100%). All three groups showed highest estimates for PPV for Down syndrome and cleft lip with or without cleft palate. This means for instance that in more than 90% of the children registered in a hospital database with a code for Down syndrome, the same child was also registered in EUROCAT with a Down syndrome diagnosis. Lowest estimates for PPV were found for microcephaly, HLHS and ASD.

**Table 4:** Estimates for PPV for congenital anomalies, diagnosed in the first year of life

Congenital anomaly	Pooled estimate for PPV							
	Group I ICD9 registries		Group II Data from health care databases		Group III Other registries		Overall pooled estimate	
	%	(95% CI)	%	(95% CI)	%	(95% CI)	%	(95% CI)
Spina bifida	49	(36–63)	88	(76–96)	79	(65–91)	Nc	
Cleft lip with or without cleft palate	86	(82–90)	92	(88–95)	94	(90–97)	Nc	
Cleft palate	64	(57–71)	93	(90–96)	85	(78–91)	Nc	
Gastroschisis			100	(94–100)	100	(93–100)	100	(98–100)
Omphalocele			96	(85–99)	71	(49–90)	Nc	
Clubfoot	83	(77–88)	99	(97–100)	83	(75–89)	Nc	
Hypoplastic left heart syndrome	74	(57–89)	74	(61–84)	63	(38–85)	71	(55–85)
Unilateral renal agenesis	60	(52–67)	94	(86–98)	46	(30–63)	Nc	
Limb reduction defects	77	(70–84)	86	(78–91)	71	(51–87)	78	(67–88)
Severe microcephaly	66	(58–72)	78	(66–88)	47	(31–64)	Nc	
VSD	78	(76–80)	98	(98–99)	79	(75–83)	Nc	
Hirschsprung disease	72	(63–80)	94	(85–98)	93	(79–99)	Nc	
ASD	16	(15–17)	41	(39–43)	15	(13–18)	Nc	
Congenital hydronephrosis	53	(48–57)	98	(96–99)	65	(59–72)	Nc	
Hypospadias	85	(82–88)	82	(77–87)	71	(65–77)	Nc	
Down syndrome	94	(91–97)	99	(96–100)	96	(92–99)	Nc	
Polydactyly	79	(74–83)	99	(97–100)	72	(63–80)	Nc	

Group I: Emilia Romagna and Valencian Region

Group II: Finland

Group III: Wales and Funen

*CI* confidence interval, *VSD* ventricular septum defect, *ASD* atrial septum defect, *Nc* overall pooled estimate was not calculated because of heterogeneity between the group estimates

Comparable to sensitivity, a low estimate for PPV could either be due to no registration of the child in EUROCAT or registration in EUROCAT with another CA code. For ASD we found that, depending on the registry, about 40 to 60% of the children with an ASD code in the hospital data were not registered in EUROCAT at all. For HLHS however, we noticed that in Valencian Region, Finland and Wales all children who had an ICD code for HLHS recorded in the hospital database, but not in the EUROCAT data, had another ICD code for a heart defect registered in EUROCAT. Related anomaly codes (same organ system) were also frequently found in EUROCAT data for limb reduction defects, unilateral renal agenesis and ASD, whereas for severe microcephaly unrelated CA codes were frequently found in the EUROCAT (data not shown).

## Discussion

In this study, we investigated the accuracy of CA coding in live born children in electronic hospital databases, by comparing the CA codes of 17 anomalies in the hospital database of linked EUROCAT cases from 11 EUROCAT registries and reference children.

We found that the proportion of the linked live births in EUROCAT registries with a CA code recorded in an electronic hospital database, varied between 57% in Northern Netherlands to 96% in Valencian Region, Spain. These proportions were comparable to proportions reported in other studies [5, 6]. There may be several reasons why, in general, a CA code in the hospital database is missing for a EUROCAT case. If the electronic hospital database includes only inpatient data, a CA code could be missing for newborns affected with a CA that does not require admission or surgery in the first year of life. Indeed, when looking at specific CAs, we found high estimates for sensitivity (> 85%) for anomalies that are visible at birth or diagnosed prenatally and require hospitalization or surgery in the first year, such as abdominal wall defects, orofacial clefts, HLHS and spina bifida, whereas anomalies that are diagnosed later in life and do not require (immediate) surgery such as VSD and ASD, showed lower or heterogeneous sensitivity. We also noticed the high sensitivity for clubfoot and hydronephrosis in Finland and Funen, Denmark, in contrast to lower sensitivity in other registries, which can be explained by the out-patient data that are present in the Finnish and Danish hospital database. Sensitivity for club foot and hydronephrosis using only the inpatient data is much lower (data not shown). Other reasons for low sensitivity due to no recording of CA codes in hospital data is when the specialist care is given in specialized hospitals outside the (regional) data coverage area. Also, if a newborn with a CA was admitted to the hospital and the admission was not related to the CA, a CA code may not be recorded for this child on that occasion in the hospital data. Low sensitivity may be also caused by the use of other (less specified or unrelated) CA codes in the hospital data, or the use of incorrect codes, such as the code for acquired hydronephrosis (N13.0) instead of congenital hydronephrosis (Q62.0).

In this study a high PPV indicates that most of the hospital cases with a CA code are correctly identified as having the anomaly (as judged by being present in EUROCAT with the same CA). A low PPV means that ‘many’ of the cases with a CA code in the hospital data did not have a CA according to EUROCAT (false positive hospital cases). PPV in our study was in general lower than sensitivity and showed more heterogeneity among the hospital databases.

Although for certain anomalies the PPV was below 85%, we frequently found other CA codes (related and unrelated) in the EUROCAT data. This means that the child was correctly identified as having a CA in the hospital database, but the diagnosis code applied by EUROCAT registry staff, after reviewing medical records from multiple sources, differed from the CA code in the hospital database. The lowest PPV was observed for ASD, which is an anomaly that presents in variable form between normal and abnormal. When an ASD code was recorded in a hospital database, the child most likely did not have a major ASD anomaly. Even when we included related CA and unrelated CA codes in the EUROCAT database, the PPV estimates remained < 60%. According to EUROCAT guidelines an ASD *secundum* should only be registered when a flow across the defect is still present 6 months after birth [15]. Because hospital databases often do not include information on related factors, such as gestational age at birth, which can differentiate between anomalies at term versus normal aspects of development in preterm births, the PPV will be lower for these anomalies.

We found that in European hospital databases the information recorded for gastroschisis, cleft lip with or without cleft palate (anomalies that are visible at birth and require surgery) and Down syndrome can be considered accurate (complete and valid), because both sensitivity and PPV estimates were high. For HLHS, spina bifida, Hirschsprung’s disease, omphalocele and cleft palate we found high sensitivity, but low or heterogeneous PPV, indicating that hospital data is complete but needs to be validated to identify the false positive cases or apply the correct diagnosis code. For the remaining anomaly subgroups in our study we found that both sensitivity and PPV were low or heterogeneous, indicating that the information in the hospital database is incomplete and of variable validity. Additional data sources are needed to capture all cases and data from hospital databases need to be validated.

Besides heterogeneity in sensitivity and PPV estimates between congenital anomaly types, we observed also heterogeneity in sensitivity and PPV estimates among the different regions. These can be due to several reasons. First, the estimates can be affected by national differences in treatment guidelines and organization of healthcare and the organization and purpose of the hospital database, including coding practices [17]. In the Northern Netherlands for instance, the hospital database system changed in the study period. The results of the Dutch hospital data showed large differences, and therefore only the data from the most recent years were used. Secondly, we standardized the hospital data in our study to a common data model, which included the abstraction of hospital data and the translation of ICD-9-CM and ICD-10 codes into CA subgroups. The standardized data used for analyses may therefore also affect sensitivity and PPV. And thirdly, EUROCAT registries who use hospital databases as a main source of case ascertainment, such as Finland and Poland, showed high estimates for sensitivity and PPV, because the hospital database was not an independent data source.

While results differ between regions, the results for the registries that linked to the same hospital database, Thames Valley, Wessex and East Midlands and South Yorkshire to the Hospital Episode Statistics, Admitted Patient Care and Tuscany and Emilia Romagna to the Scheda di Dimissione Ospedaliera, are comparable.

### Strengths

This is the first study to investigate the accuracy of hospital coding of CA in several European hospital databases, using EUROCAT as a gold standard. EUROCAT registries are high quality multiple source registries, that register and code CA according to the EUROCAT guidelines and use the EUROCAT Data Management Software for data validation, standardization and transmission to the Central Registry [1]. In this study we applied strict definitions on the codes to identify CA and used these definitions both on EUROCAT and hospital data. Standardization and analysis scripts were written centrally and applied by the individual registries, ensuring robust analysis of the EUROCAT and hospital databases.

### Limitations

We could not analyze all CA subgroups but focused on a limited number of anomalies and on diagnoses made in the first year of life. Reporting restrictions for small numbers in the Netherlands, England and Denmark limited the interpretation of the results of these registries. It was not possible to calculate PPV for all registries. Estimates of PPV for registries with access to a sample of the reference population were subject to small sample size errors and therefore we decided only to include registries with access to the full reference population. The study was only able to compare the codes for live births as pregnancies that result in termination because of a prenatal diagnosis for fetal anomalies are missing in hospital discharge databases.

### Conclusion

In conclusion, we found that European hospital databases accurately record only a limited number of anomalies in live born children, such as cleft lip with or without cleft palate, gastroschisis and Down syndrome. CAs that do not require hospitalization or surgery are often underreported in hospital discharge databases. Also, hospital databases have limited information or codes to identify pregnancies that result in termination because of a prenatal diagnosis for fetal anomalies, which is particularly relevant for anomalies with a high termination rate, such as spina bifida, certain heart anomalies and chromosomal anomalies [18]. To improve the quality of CA coding in electronic health care databases, we recommend using extended versions of the ICD coding system so that the most specific codes can be reported. Also we advise to allow revision of the previously entered codes by more experienced doctors or coders or after results of diagnostic examinations [19]. Outpatient data should be used if available to improve completeness. To optimize the use of electronic hospital databases and obtain the maximum amount of accurate information, the application of a validated algorithm using a set of codes (for instance including procedures and cross-referencing multiple sources) is recommended. In regions where a CA registry exists, hospital data could be an additional source for active searching of CA cases, not otherwise reported by the CA registry [20, 21]. Also, the development of such an algorithm may be useful for CA registries in order to ensure that cases/clinical diagnoses identified do not include differential or unconfirmed diagnoses [21, 22].

Researchers using electronic hospital databases, should collaborate with coders from these hospital databases to be informed of coding practices and on the specific codes that are used for specific anomalies. Validated algorithms should be used to identify congenital anomalies. As an aid to interpretation of the results and for quality improvement we advise to discuss the results with people working with the healthcare databases [19].

Even though electronic health care databases can be used as an additional source for CA registries, electronic health care databases alone cannot replace congenital anomaly registries. CA registries where experts validate and code the CA based on all available information are still the most appropriate data source to monitor the prevalence of CAs, evaluate health care policies and study possible risk factors.

## Supplementary Information

Below is the link to the electronic supplementary material.

Supplementary file1 (PDF 138 KB)

Supplementary file2 (PDF 841 KB)

Supplementary file3 (PDF 813 KB)
