# Supplementary material for: Accuracy of congenital anomaly coding in live birth children recorded in European health care databases, a EUROlinkCAT study
Source: Eur J Epidemiol. 2023 Feb 18;38(3):325–34. doi: 10.1007/s10654-023-00971-z (PMC10033551; doi:10.1007/s10654-023-00971-z)
Supplement: Supplementary file 1 — Supplementary file1 (PDF 138 KB) [file 10654_2023_971_MOESM1_ESM.pdf]

Supplementary table A

ICD9-CM and ICD10 codes for the seventeen anomalies included in study.

Anomaly	ICD9-CM	ICD10
**Detectable at birth**		
- Spina bifida	741	Q05
- Cleft lip +/-cleft palate	749.1 , 749.2	Q36, Q37
- Cleft palate	749.0	Q35
- Gastroschisis	756.73	Q793
- Omphalocele	756.72	Q792
- Clubfoot	754.51	Q660
**High prenatal detection rate**		
- Hypoplastic left heart syndrome	746.7	Q234
- Unilateral renal agenesis	753.0	Q600
- Limb reduction defects	755.2-755.3	Q71-Q73
**Diagnosed after discharge from maternity unit**		
- Severe microcephaly	742.1	Q02
- Ventricular Septum Defects	745.4	Q210
- Hirschsprung's disease	751.3	Q431
**Grey zone between normal and abnormal**		
- Atrial Septum Defects	745.5	Q211
- Hydronephrosis	753.20	Q620
- Hypospadias	752.61	Q54
**Chromosomal anomaly**		
- Down syndrome	758.0	Q90
**Mild anomaly**		
- Polydactyly	755.0	Q69

MK Bakker *et al*. Accuracy of congenital anomaly coding in live birth children recorded in European health care databases, a EUROlinkCAT study
