# Supplementary material for: Accuracy of congenital anomaly coding in live birth children recorded in European health care databases, a EUROlinkCAT study
Source: Eur J Epidemiol. 2023 Feb 18;38(3):325–34. doi: 10.1007/s10654-023-00971-z (PMC10033551; doi:10.1007/s10654-023-00971-z)

Anomalies detectable at birth

Estimates for sensitivity per registry, pooled estimates per group and overall pooled estimate.

NA indicates that the number and/or the estimate cannot be reported because of release restrictions for small numbers.

CAROBB: Thames Valley
WANDA: Wessex
EMSCYAR: East Midlands and South Yorkshire

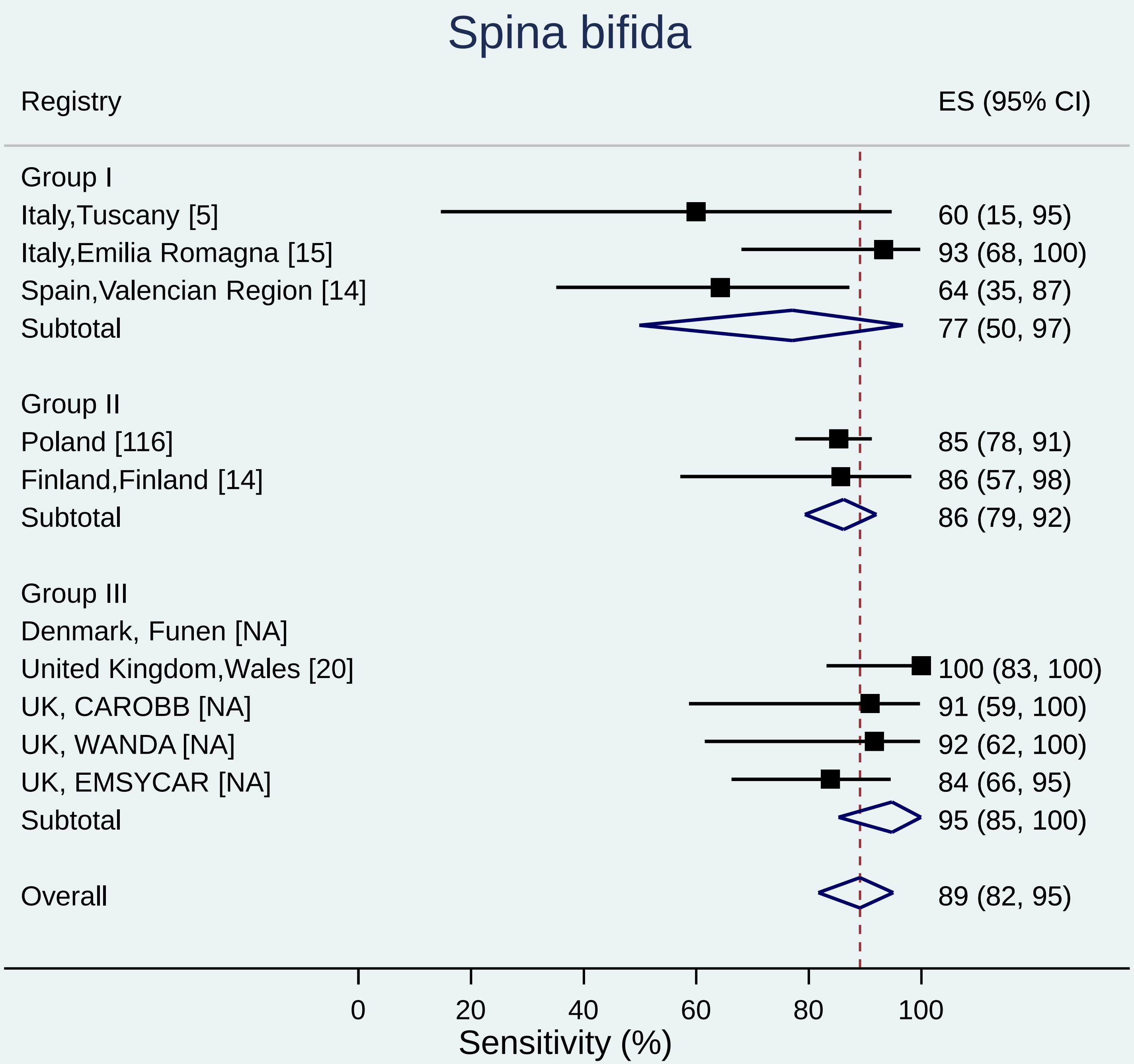

Supplement B
MK Bakker et al. Accuracy of congenital anomaly coding in live birth children recorded in European health care databases, a EUROlinkCAT study

Anomalies detectable at birth

Estimates for sensitivity per registry, pooled estimates per group and overall pooled estimate.

NA indicates that the number and/or the estimate cannot be reported because of release restrictions for small numbers.

CAROBB: Thames Valley
WANDA: Wessex
EMSCYAR: East Midlands and South Yorkshire

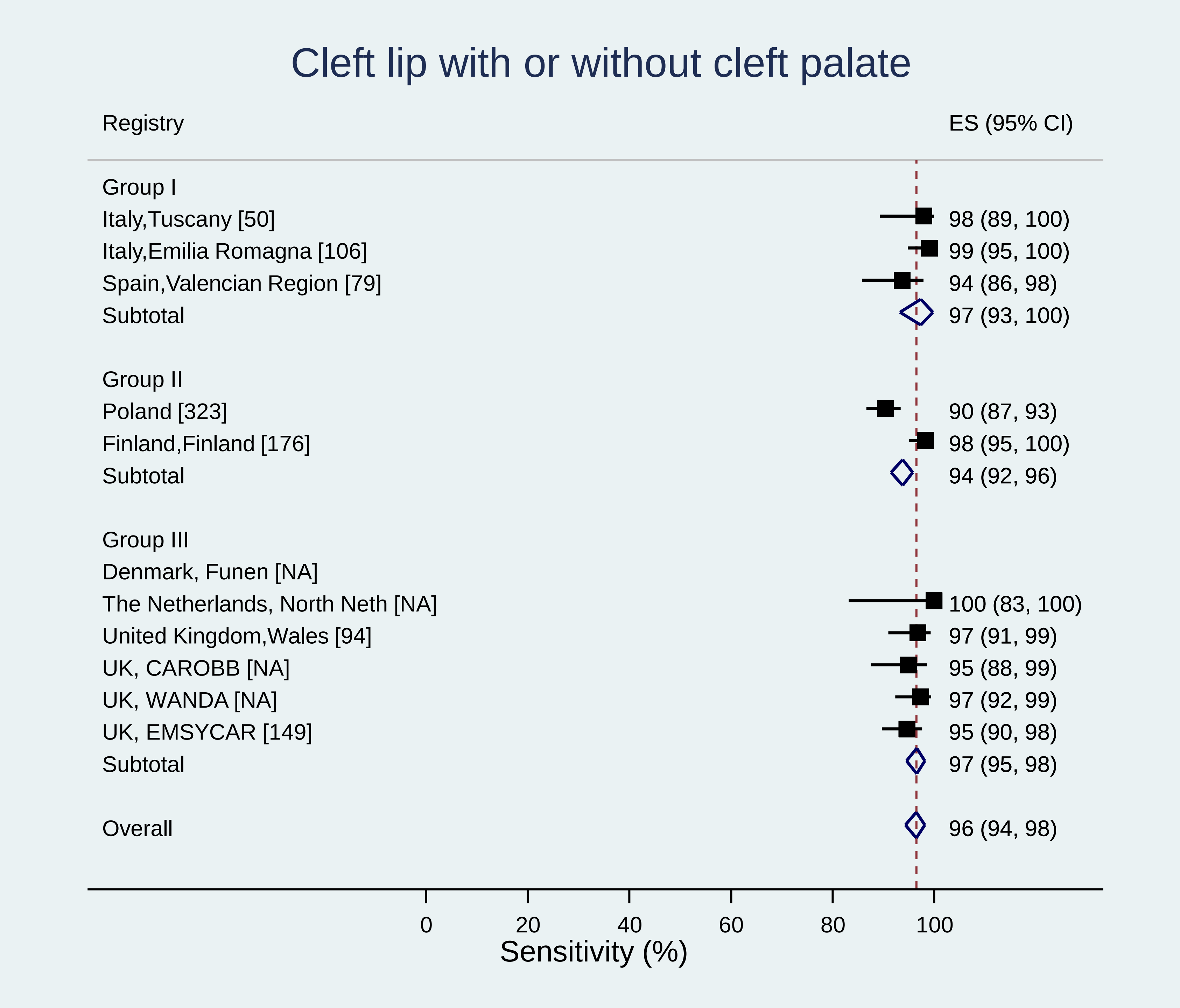

Anomalies detectable at birth

Estimates for sensitivity per registry, pooled estimates per group and overall pooled estimate.

NA indicates that the number and/or the estimate cannot be reported because of release restrictions for small numbers.

CAROBB: Thames Valley
WANDA: Wessex
EMSCYAR: East Midlands and South Yorkshire

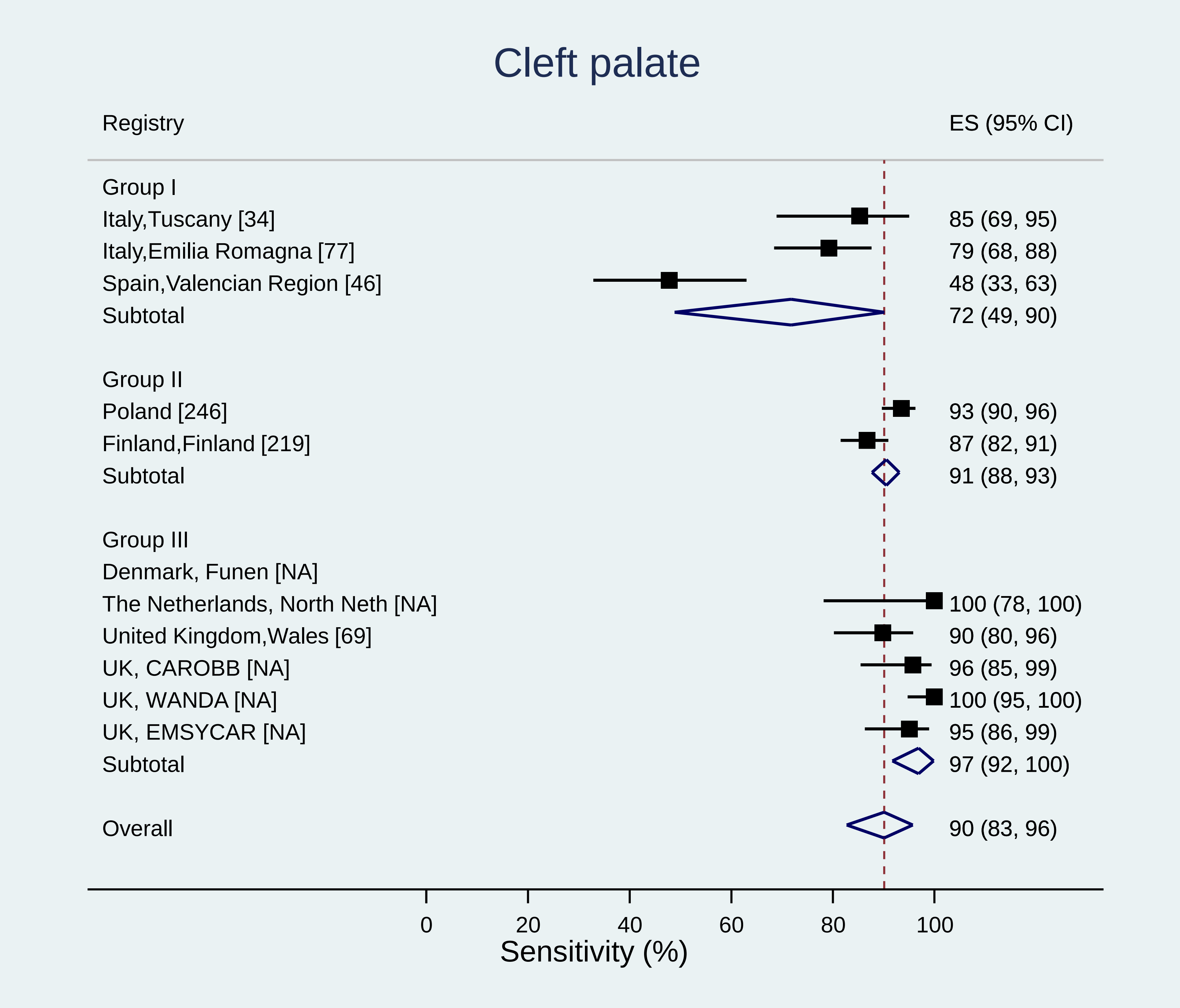

Anomalies detectable at birth

Estimates for sensitivity per registry, pooled estimates per group and overall pooled estimate.

NA indicates that the number and/or the estimate cannot be reported because of release restrictions for small numbers.

CAROBB: Thames Valley
WANDA: Wessex
EMSCYAR: East Midlands and South Yorkshire

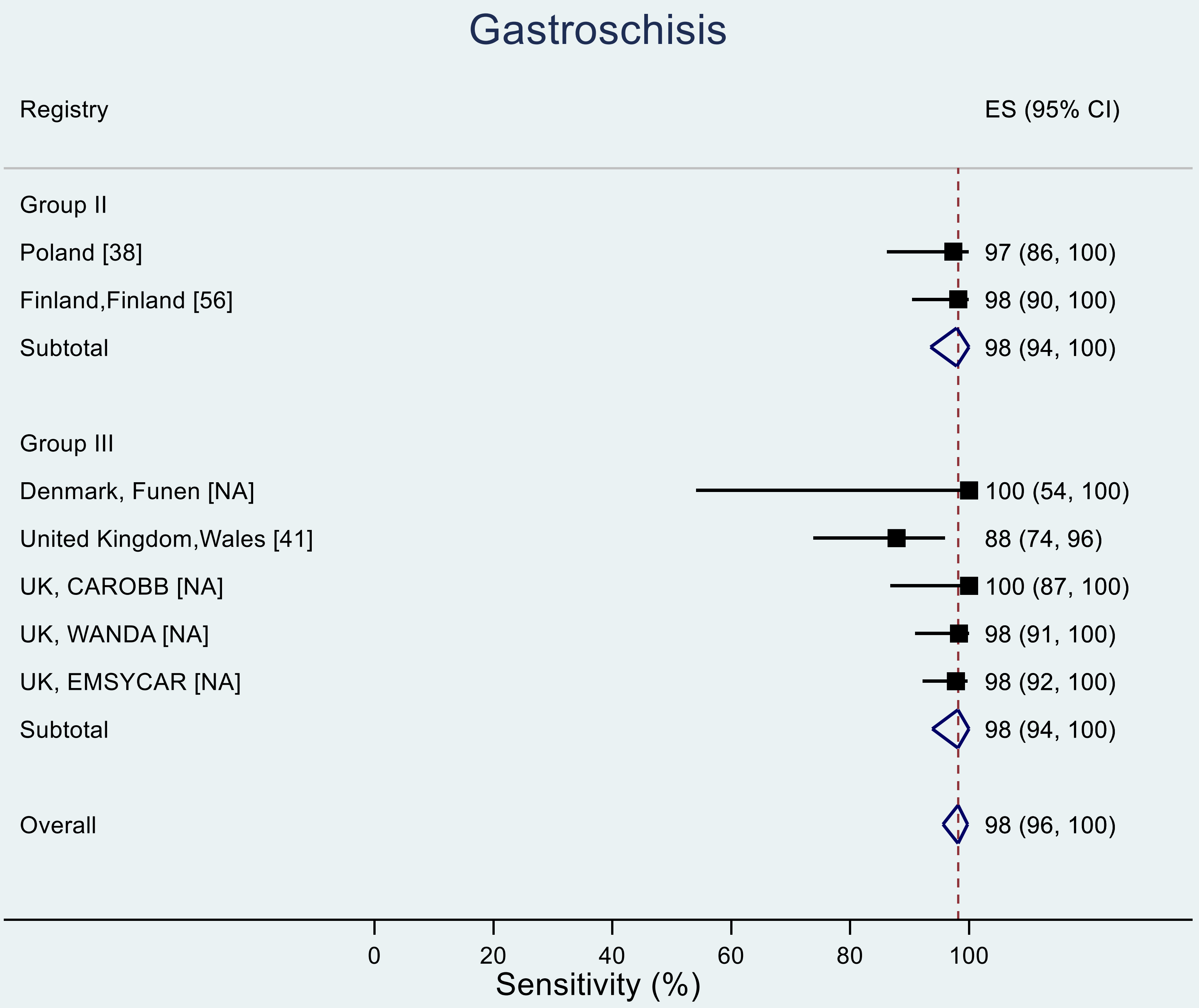

Anomalies detectable at birth

Estimates for sensitivity per registry, pooled estimates per group and overall pooled estimate.

NA indicates that the number and/or the estimate cannot be reported because of release restrictions for small numbers.

CAROBB: Thames Valley
WANDA: Wessex
EMSCYAR: East Midlands and South Yorkshire

Omphalocele

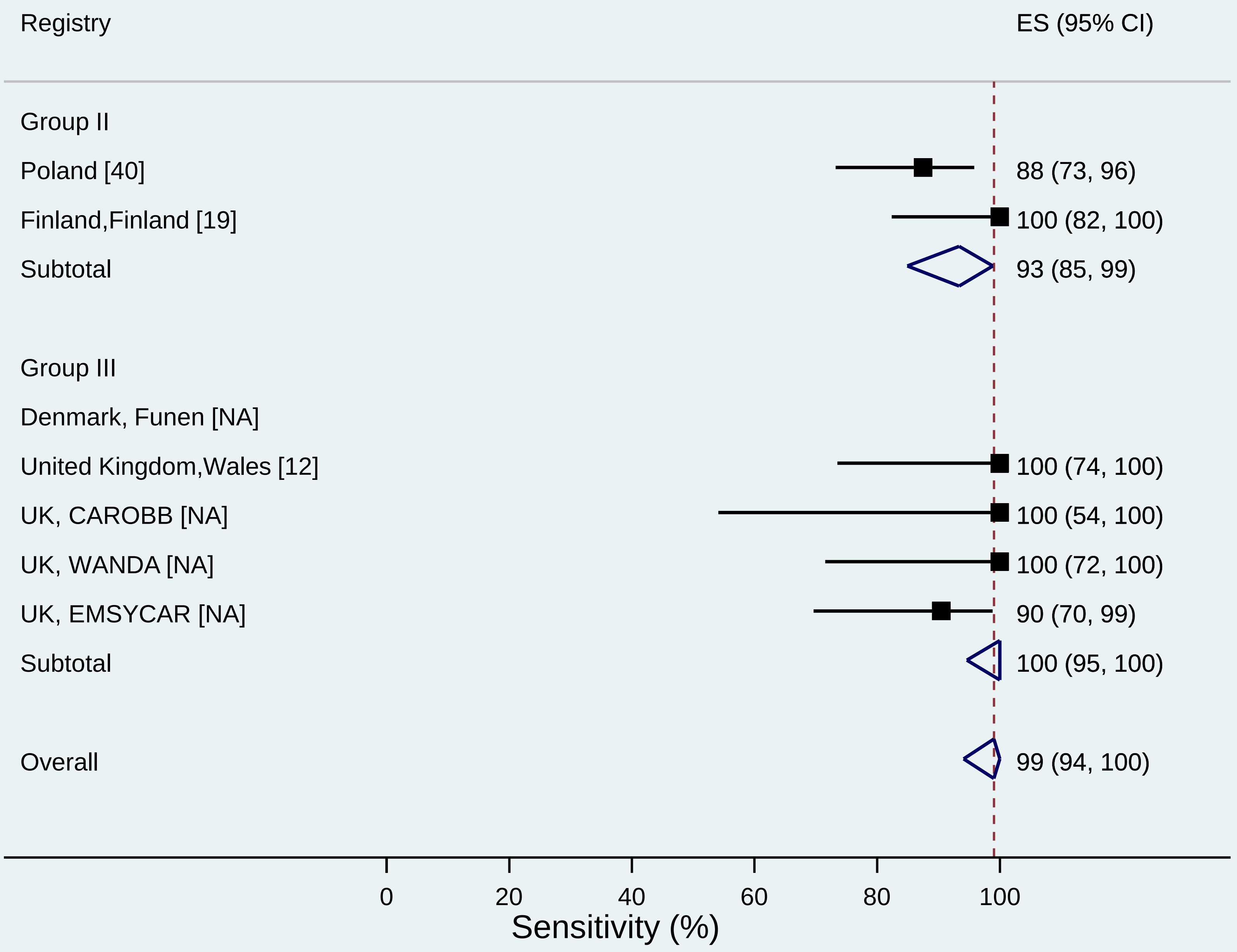

Anomalies detectable at birth

Estimates for sensitivity per registry, pooled estimates per group.

NA indicates that the number and/or the estimate cannot be reported because of release restrictions for small numbers.

CAROBB: Thames Valley
WANDA: Wessex
EMSCYAR: East Midlands and South Yorkshire

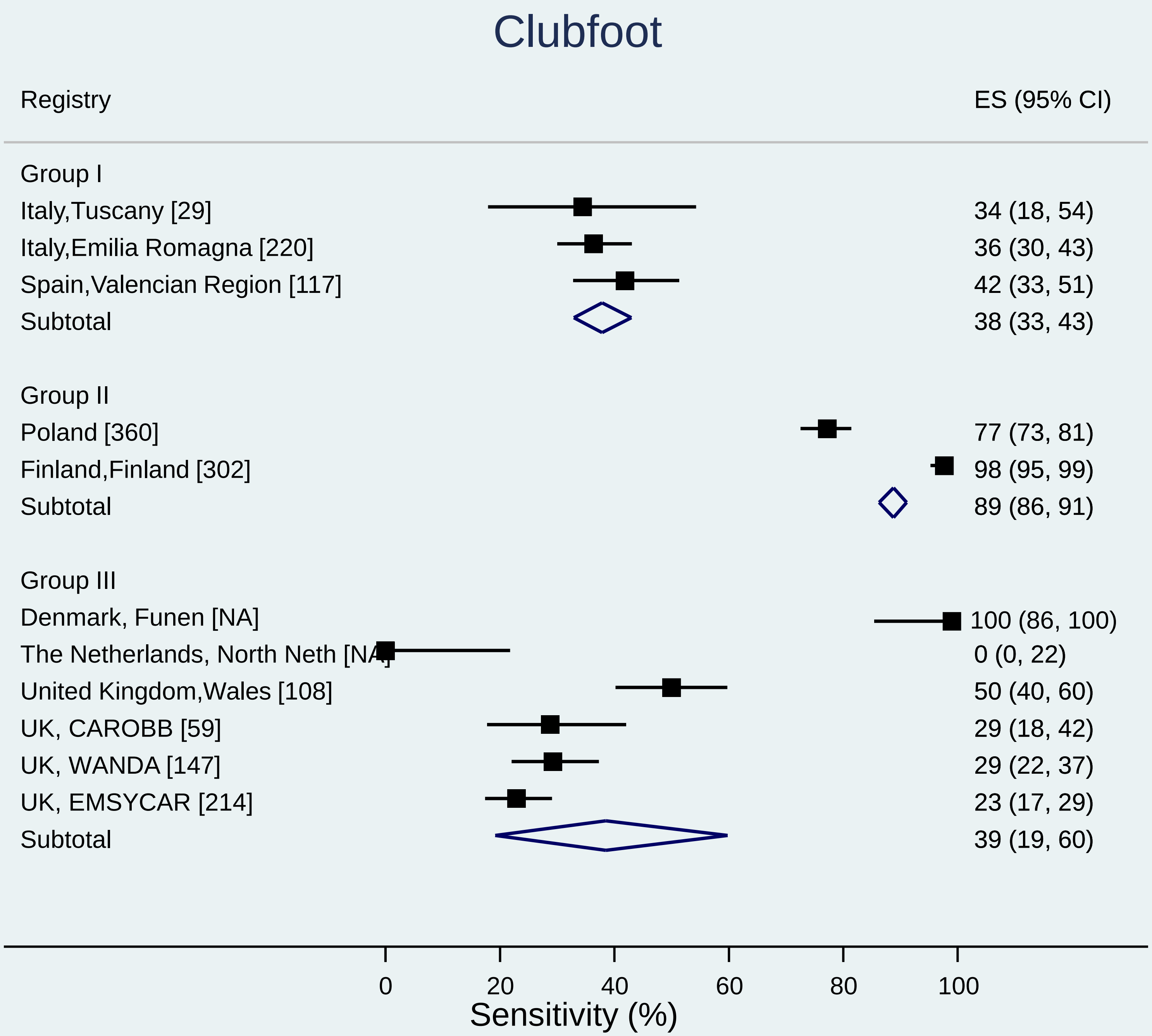

Anomalies with a high prenatal detection rate

Estimates for sensitivity per registry, pooled estimates per group and overall pooled estimate.

NA indicates that the number and/or the estimate cannot be reported because of release restrictions for small numbers.

CAROBB: Thames Valley
WANDA: Wessex
EMSCYAR: East Midlands and South Yorkshire

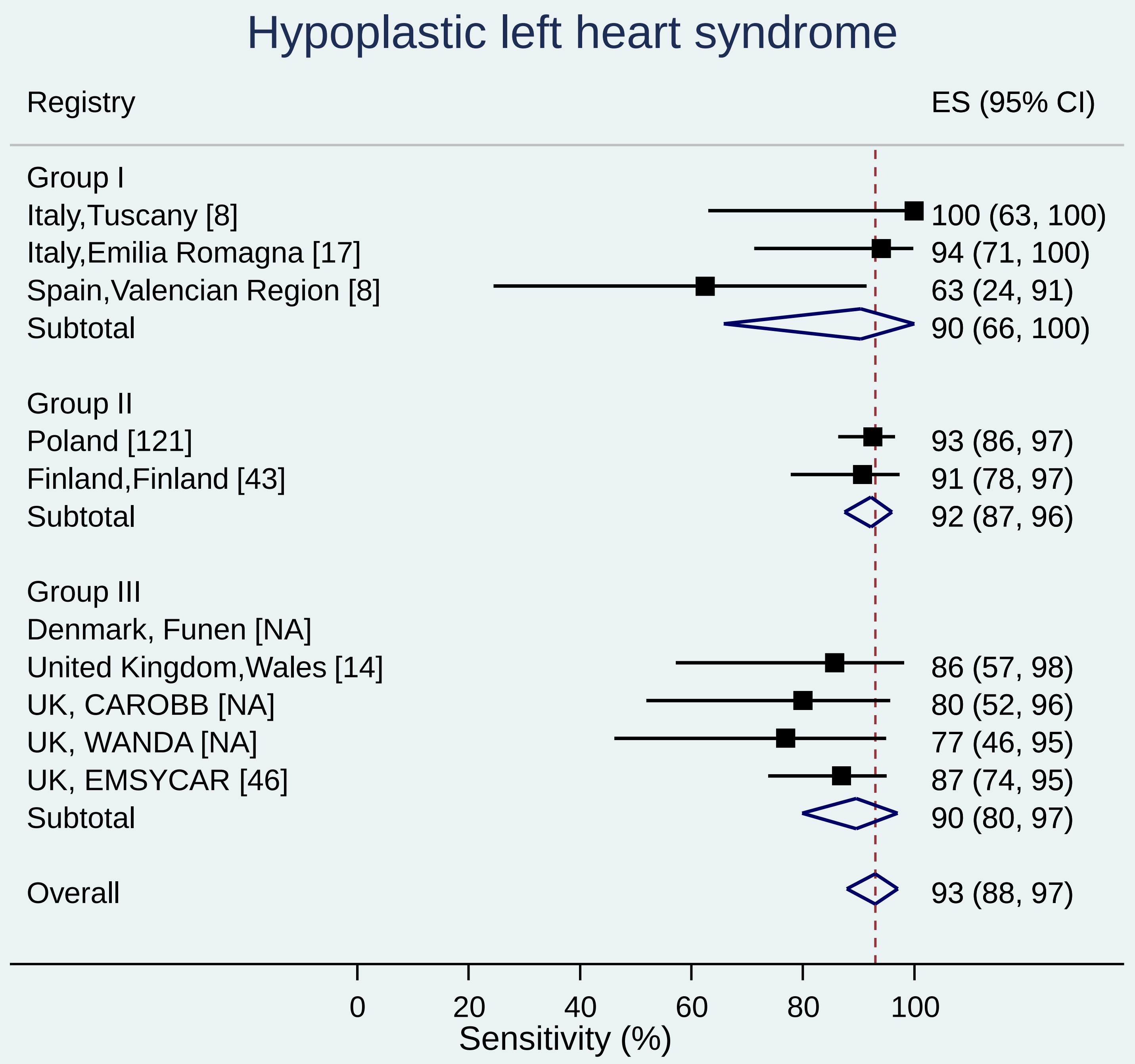

Anomalies with a high prenatal detection rate

Estimates for sensitivity per registry, pooled estimates per group.

NA indicates that the number and/or the estimate cannot be reported because of release restrictions for small numbers.

CAROBB: Thames Valley
WANDA: Wessex
EMSCYAR: East Midlands and South Yorkshire

Unilateral renal agenesis

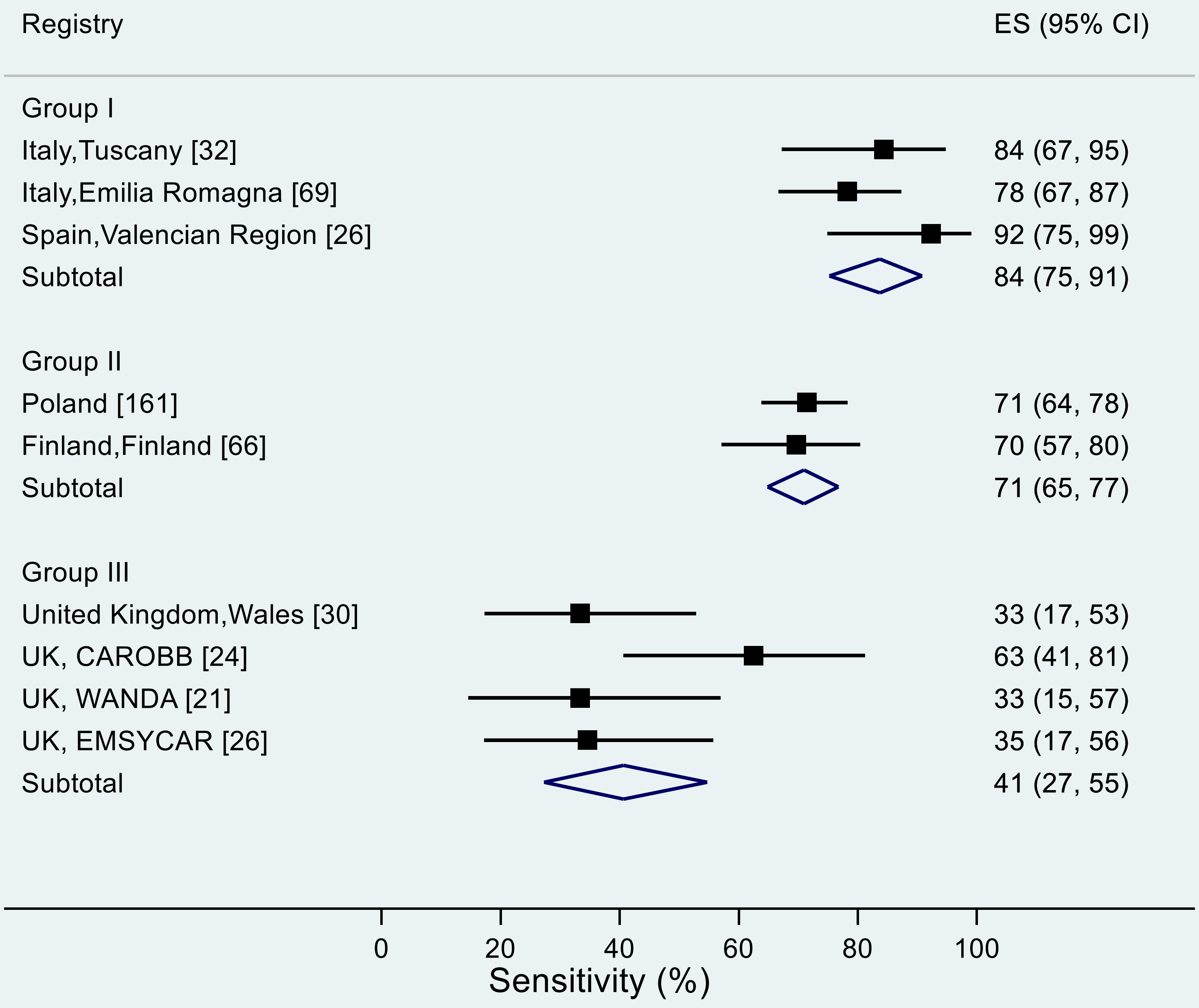

Anomalies with a high prenatal detection rate

Estimates for sensitivity per registry, pooled estimates per group.

NA indicates that the number and/or the estimate cannot be reported because of release restrictions for small numbers.

CAROBB: Thames Valley
WANDA: Wessex
EMSCYAR: East Midlands and South Yorkshire

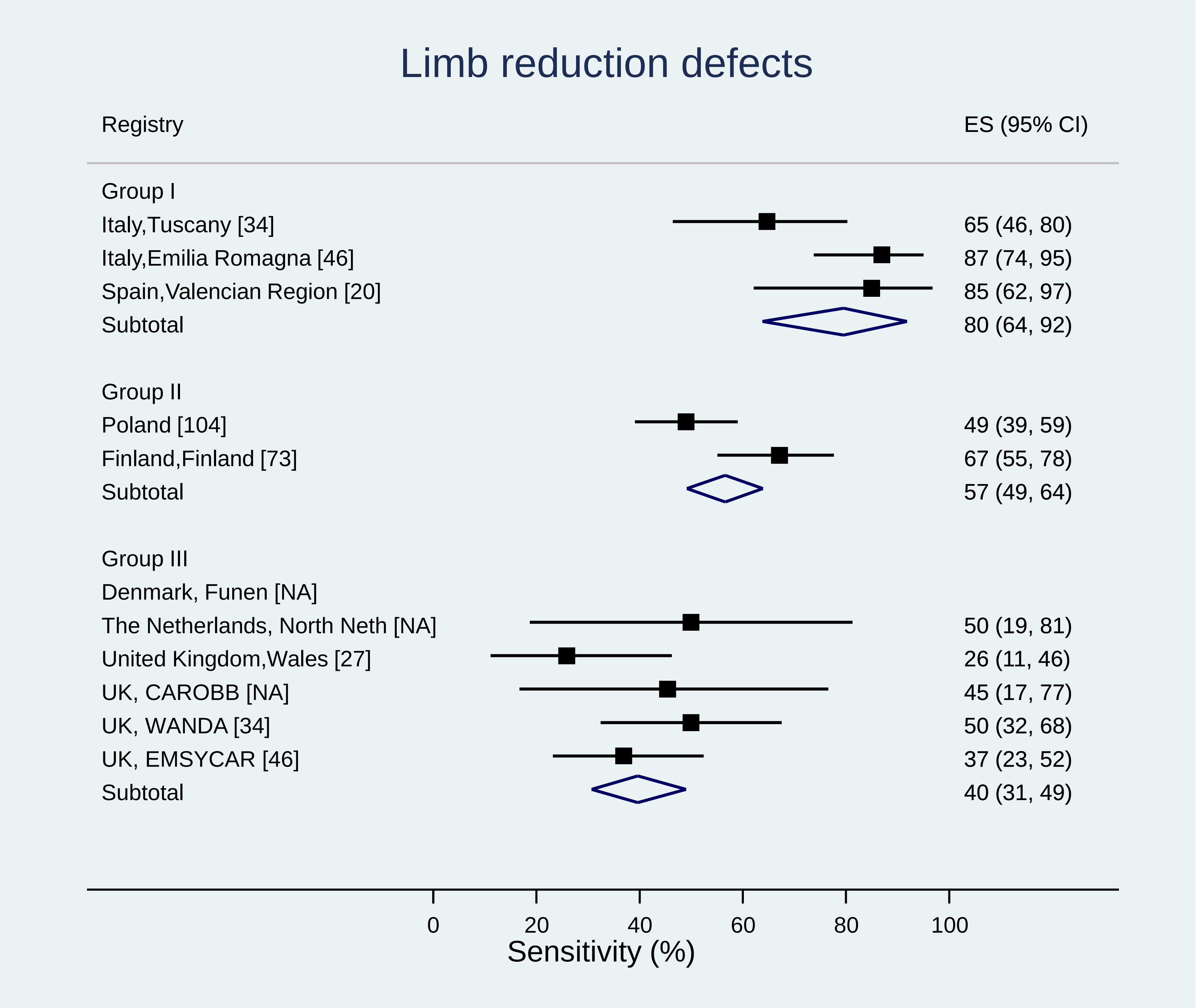

Anomalies usually diagnosed after discharge from the maternity unit

Estimates for sensitivity per registry, pooled estimates per group.

NA indicates that the number and/or the estimate cannot be reported because of release restrictions for small numbers.

CAROBB: Thames Valley
WANDA: Wessex
EMSCYAR: East Midlands and South Yorkshire

Microcephaly

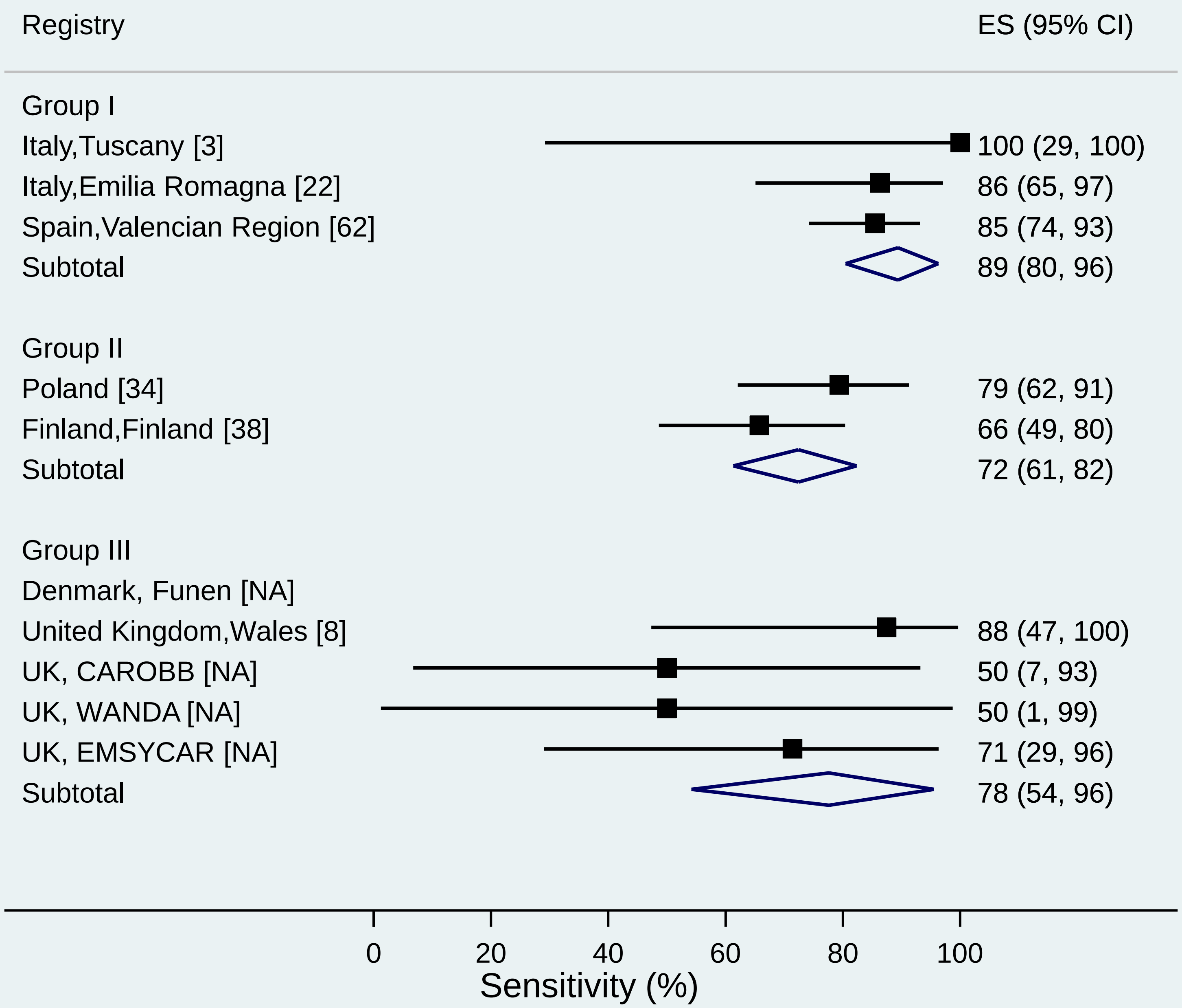

Anomalies usually diagnosed after discharge from the maternity unit

Estimates for sensitivity per registry, pooled estimates per group and overall pooled estimate.

NA indicates that the number and/or the estimate cannot be reported because of release restrictions for small numbers.

CAROBB: Thames Valley
WANDA: Wessex
EMSCYAR: East Midlands and South Yorkshire

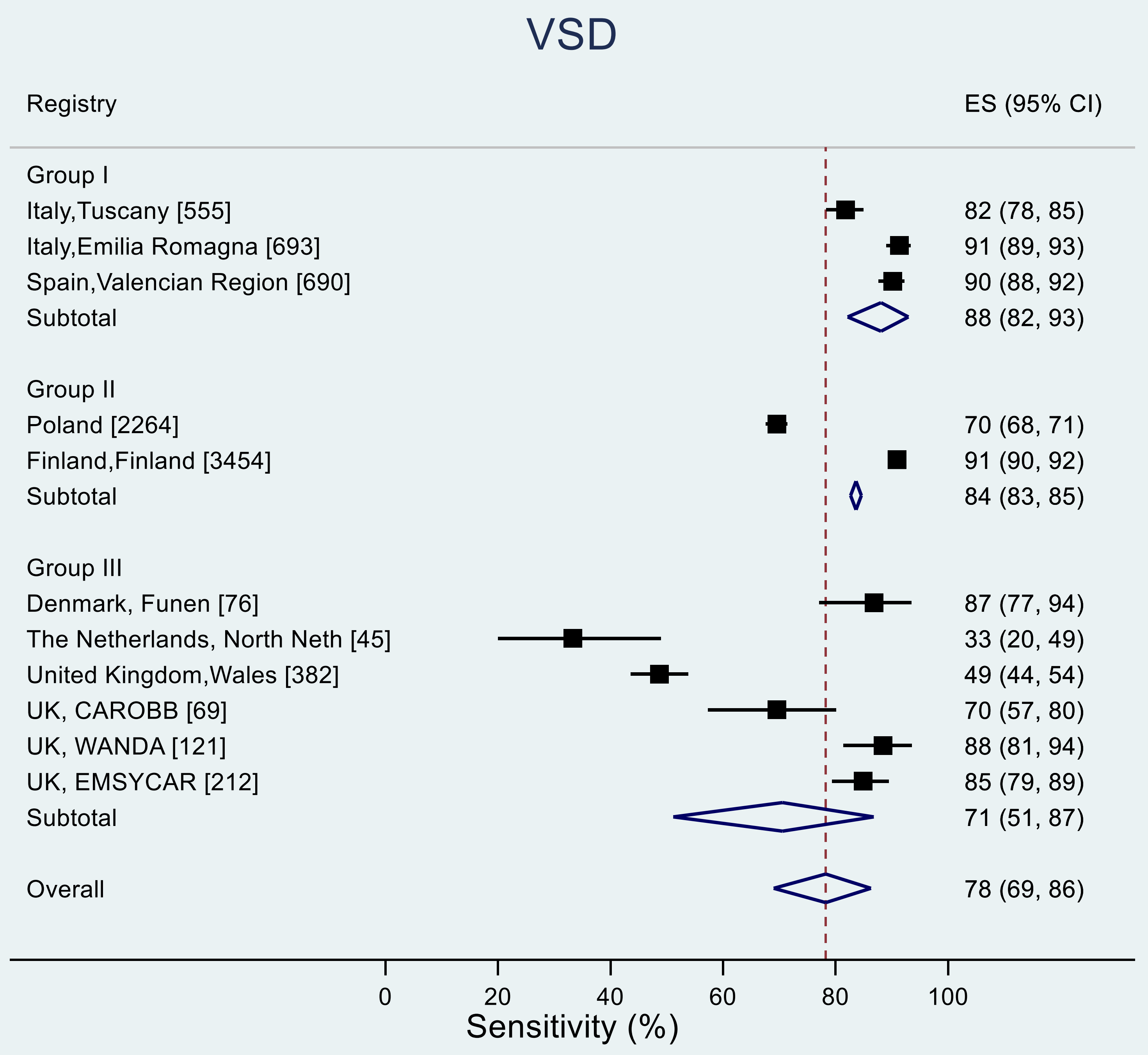

Anomalies usually diagnosed after discharge from the maternity unit

Estimates for sensitivity per registry, pooled estimates per group and overall pooled estimate.

NA indicates that the number and/or the estimate cannot be reported because of release restrictions for small numbers.

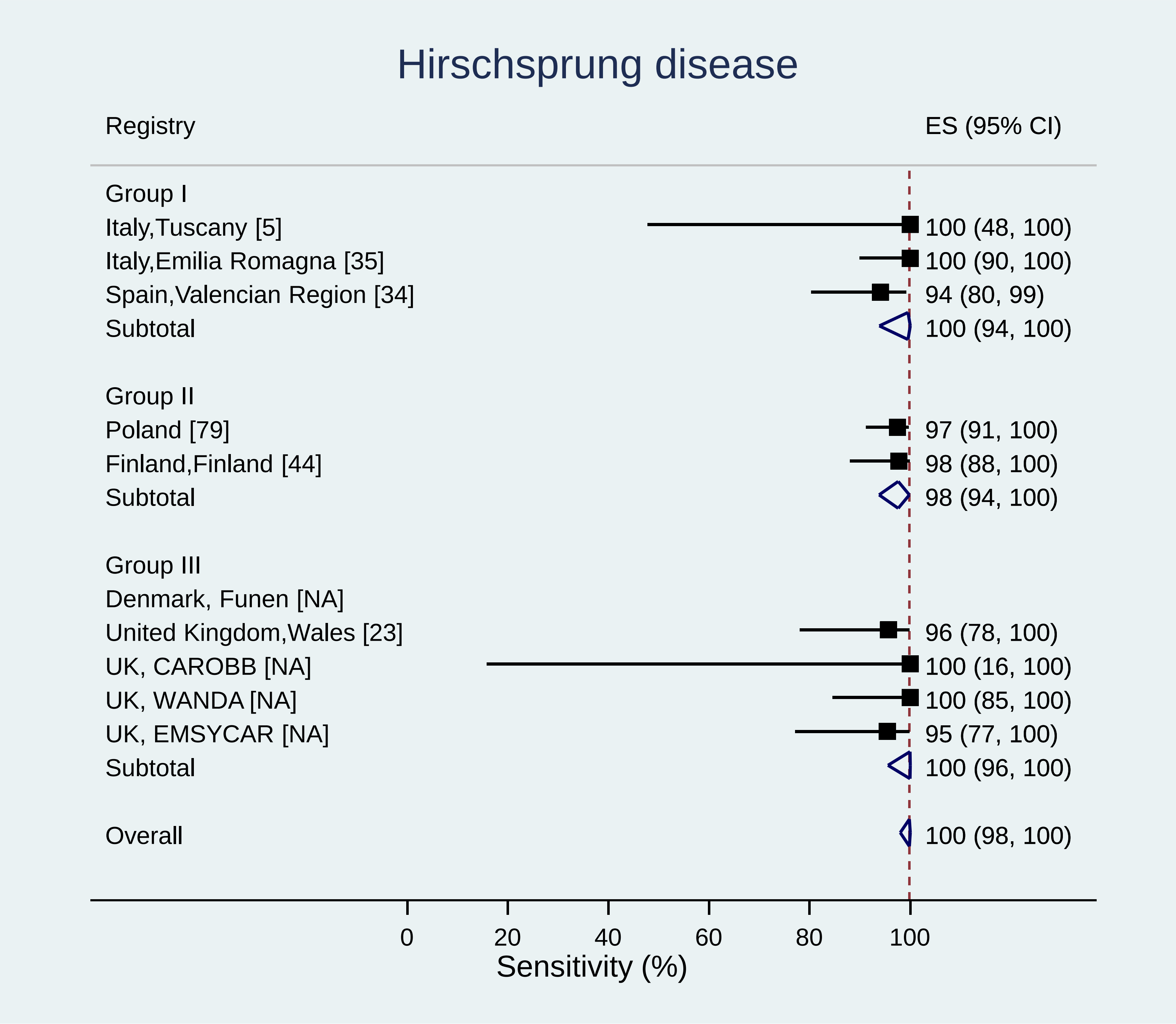

Anomalies that present in variable form between normal and abnormal

Estimates for sensitivity per registry, pooled estimates per group and overall pooled estimate.

NA indicates that the number and/or the estimate cannot be reported because of release restrictions for small numbers.

CAROBB: Thames Valley
WANDA: Wessex
EMSCYAR: East Midlands and South Yorkshire

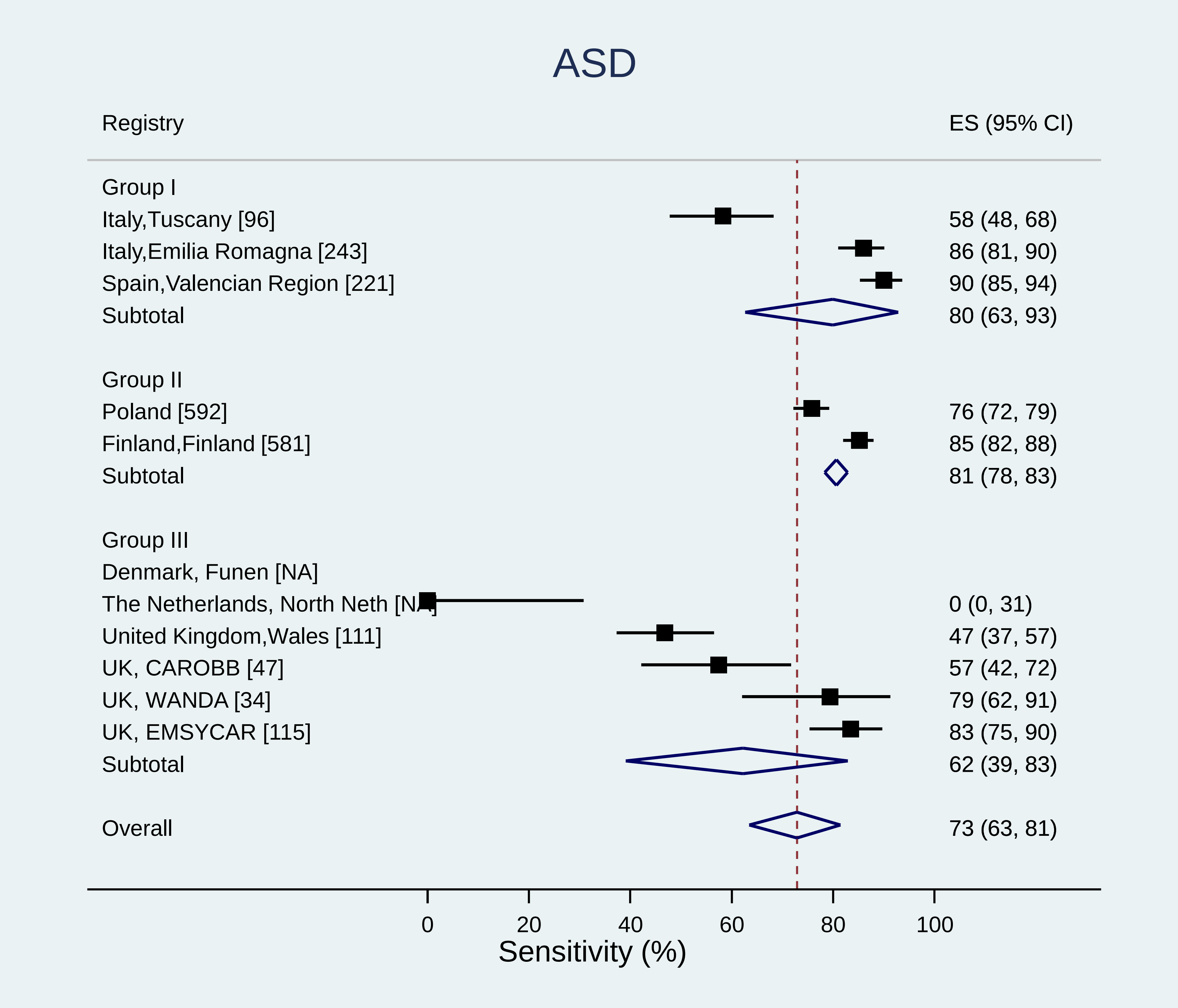

Anomalies that present in variable form between normal and abnormal

Estimates for sensitivity per registry, pooled estimates per group.

NA indicates that the number and/or the estimate cannot be reported because of release restrictions for small numbers.

CAROBB: Thames Valley
WANDA: Wessex
EMSCYAR: East Midlands and South Yorkshire

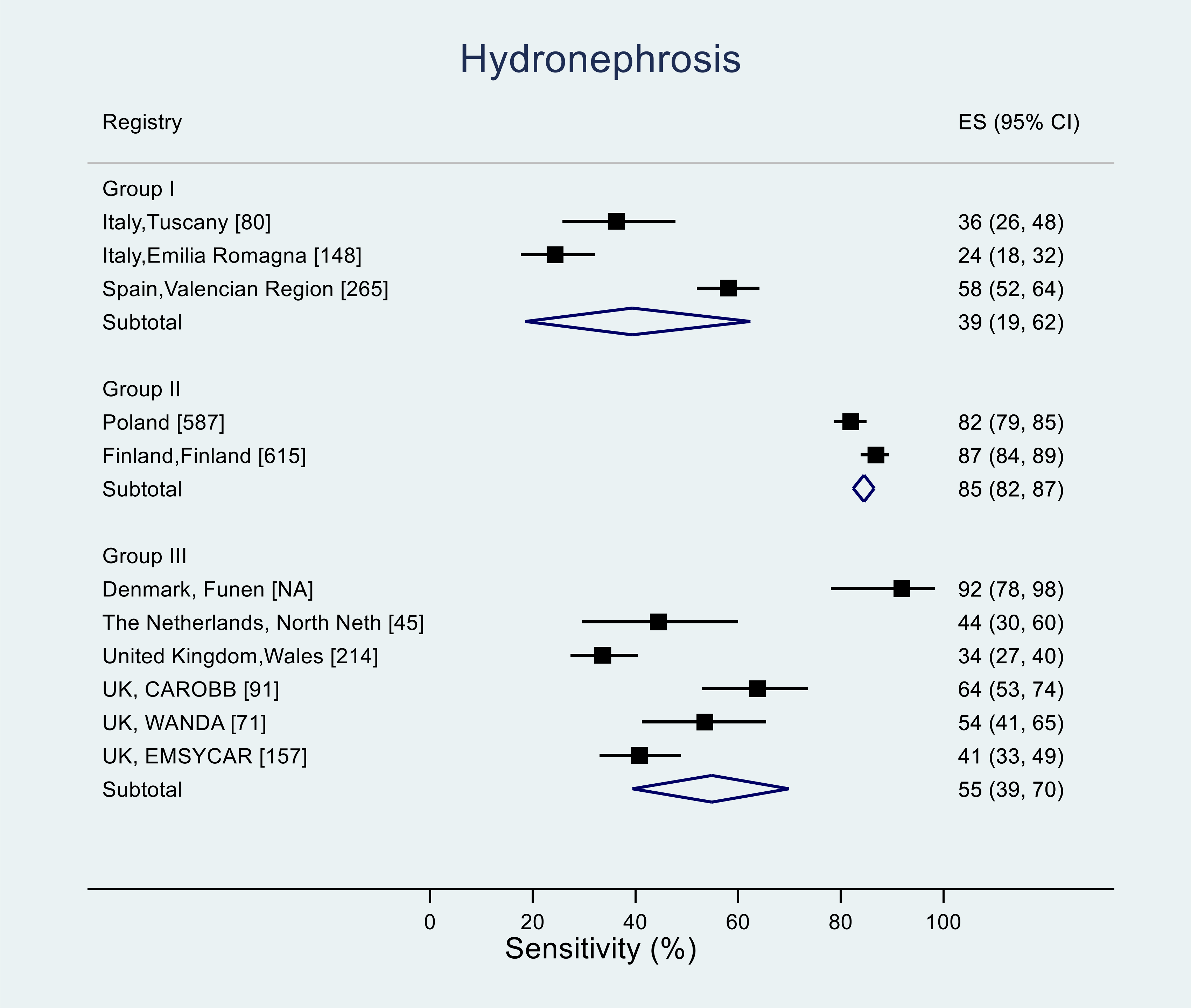

Anomalies that present in variable form between normal and abnormal

Estimates for sensitivity per registry, pooled estimates per group and overall pooled estimate.

NA indicates that the number and/or the estimate cannot be reported because of release restrictions for small numbers.

CAROBB: Thames Valley
WANDA: Wessex
EMSCYAR: East Midlands and South Yorkshire

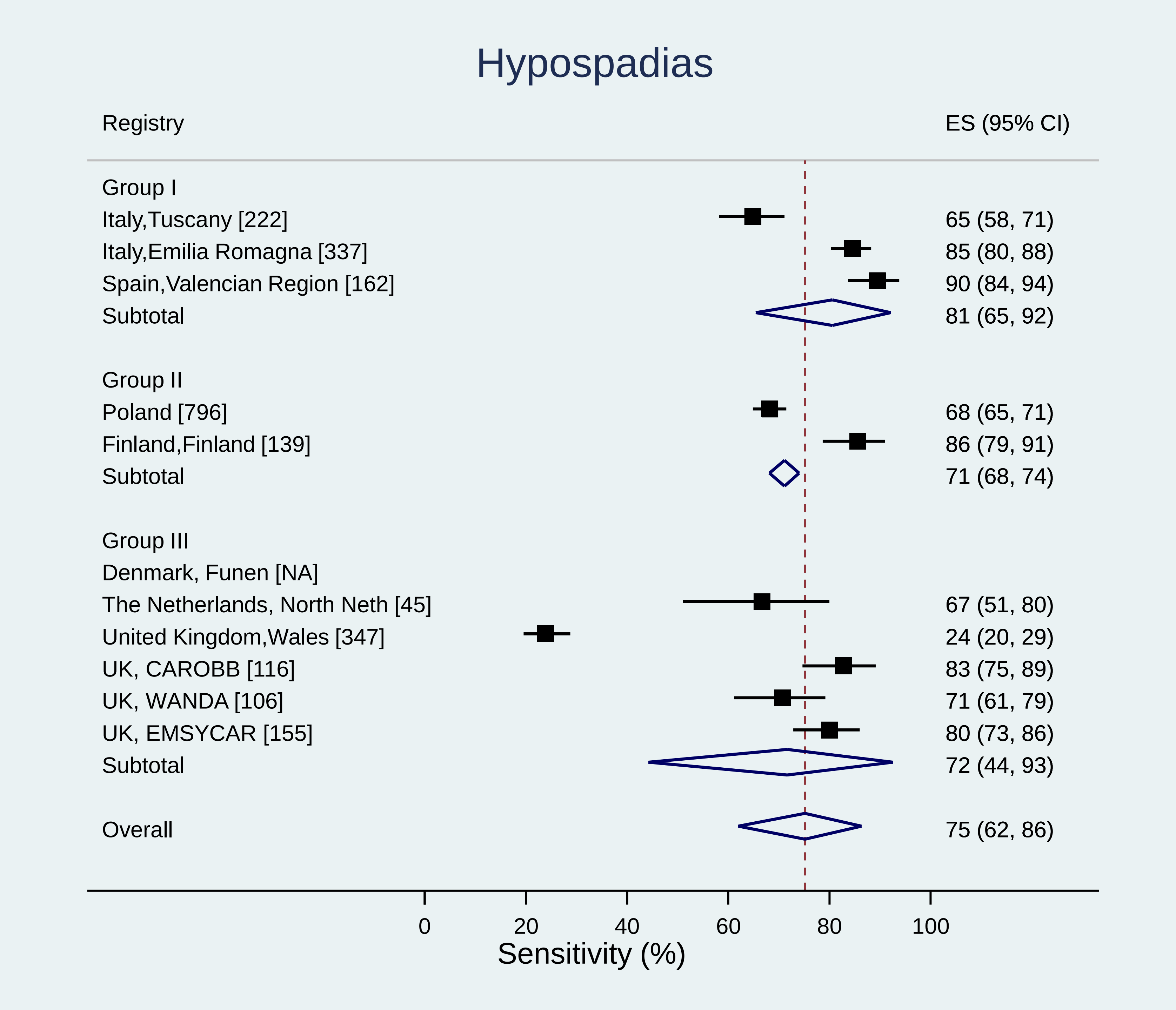

Chromosomal anomaly

NA indicates that the number and/or the estimate cannot be reported because of release restrictions for small numbers.

Estimates for sensitivity per registry, pooled estimates per group and overall pooled estimate.

CAROBB: Thames Valley
WANDA: Wessex
EMSCYAR: East Midlands and South Yorkshire

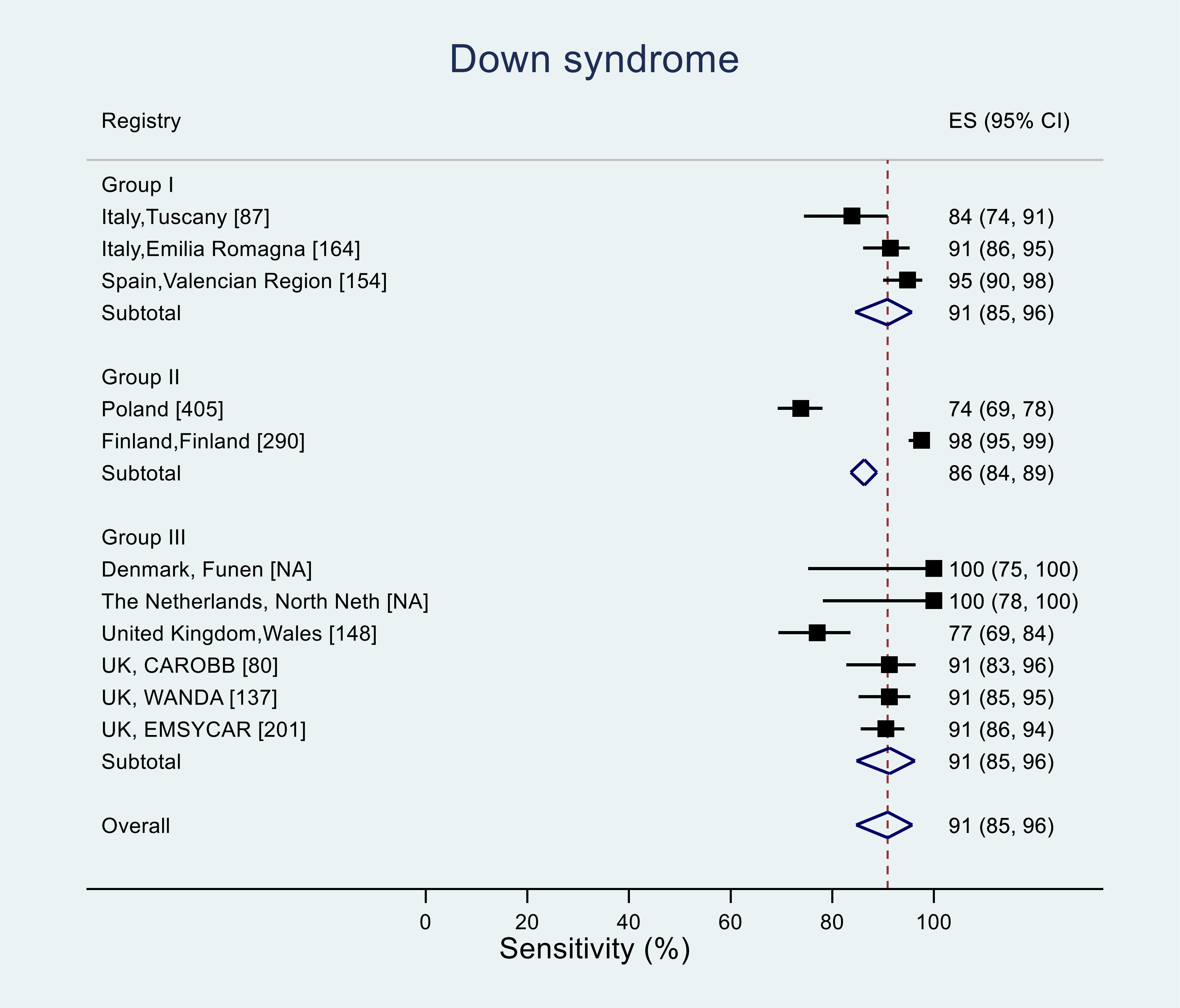

Mild anomaly

NA indicates that the number and/or the estimate cannot be reported because of release restrictions for small numbers.

Estimates for sensitivity per registry, pooled estimates per group and overall pooled estimate.

CAROBB: Thames Valley
WANDA: Wessex
EMSCYAR: East Midlands and South Yorkshire

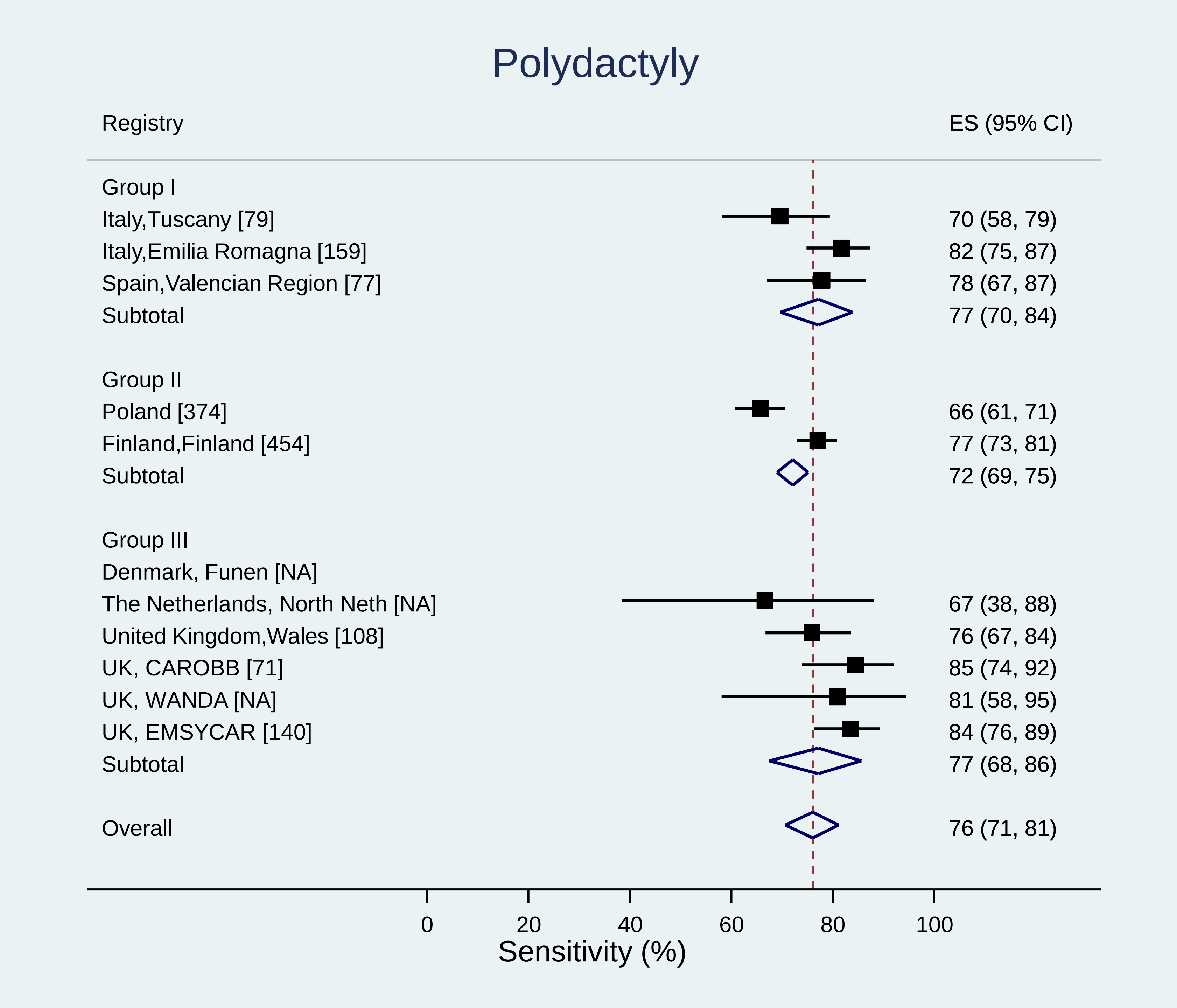

Supplement: Supplementary file 2 — Supplementary file2 (PDF 841 KB) [file 10654_2023_971_MOESM2_ESM.pdf]
