# Supplementary material for: Accuracy of congenital anomaly coding in live birth children recorded in European health care databases, a EUROlinkCAT study
Source: Eur J Epidemiol. 2023 Feb 18;38(3):325–34. doi: 10.1007/s10654-023-00971-z (PMC10033551; doi:10.1007/s10654-023-00971-z)

Anomalies detectable at birth

Estimates for PPV per registry, pooled estimates per group.

NA indicates that the number and/or the estimate cannot be reported because of release restrictions for small numbers.

Spina bifida

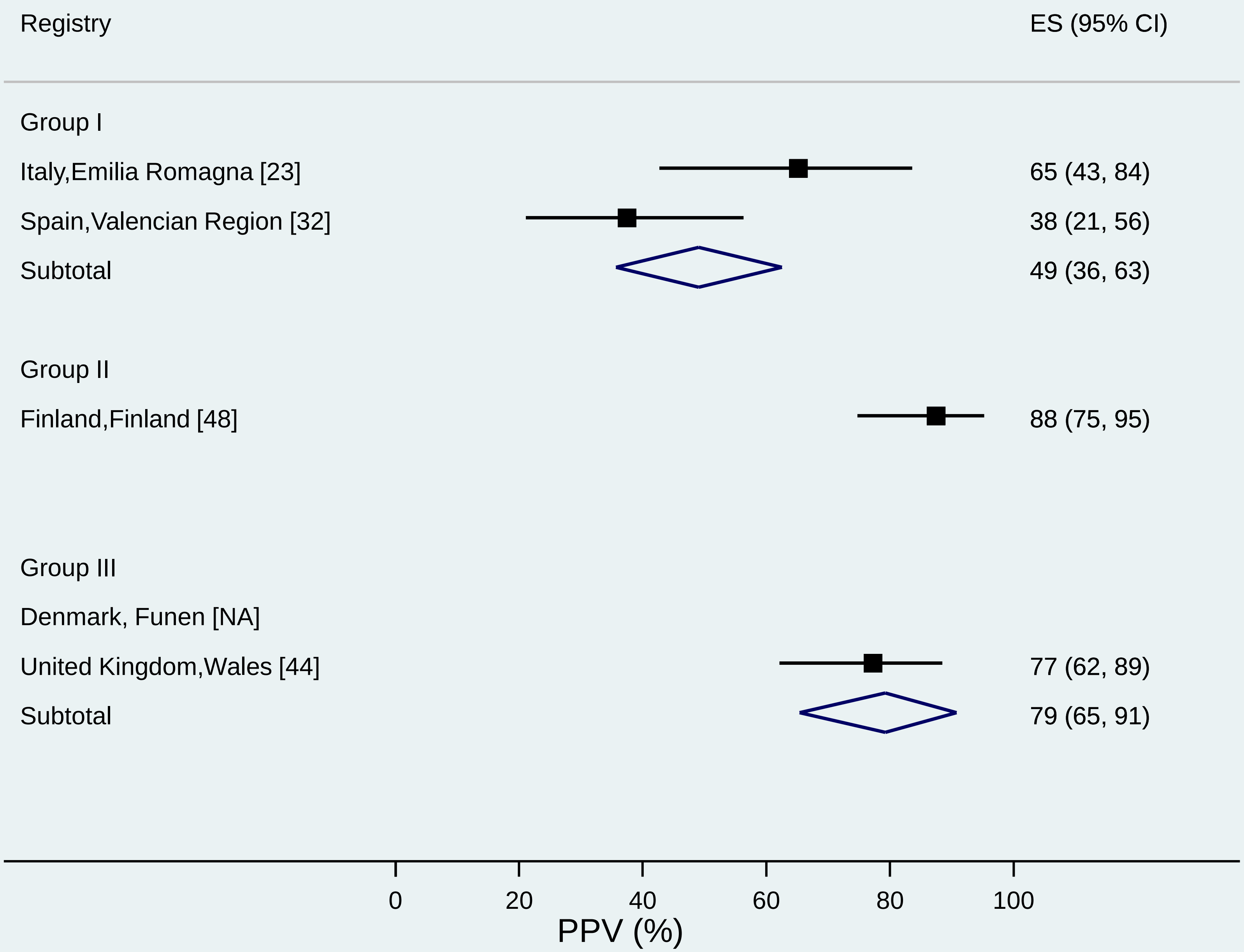

Supplement C
MK Bakker et al. Accuracy of congenital anomaly coding in live birth children recorded in European health care databases, a EUROlinkCAT study

Anomalies detectable at birth

Estimates for PPV per registry, pooled estimates per group.

NA indicates that the number and/or the estimate cannot be reported because of release restrictions for small numbers.

Cleft lip with or without cleft palate

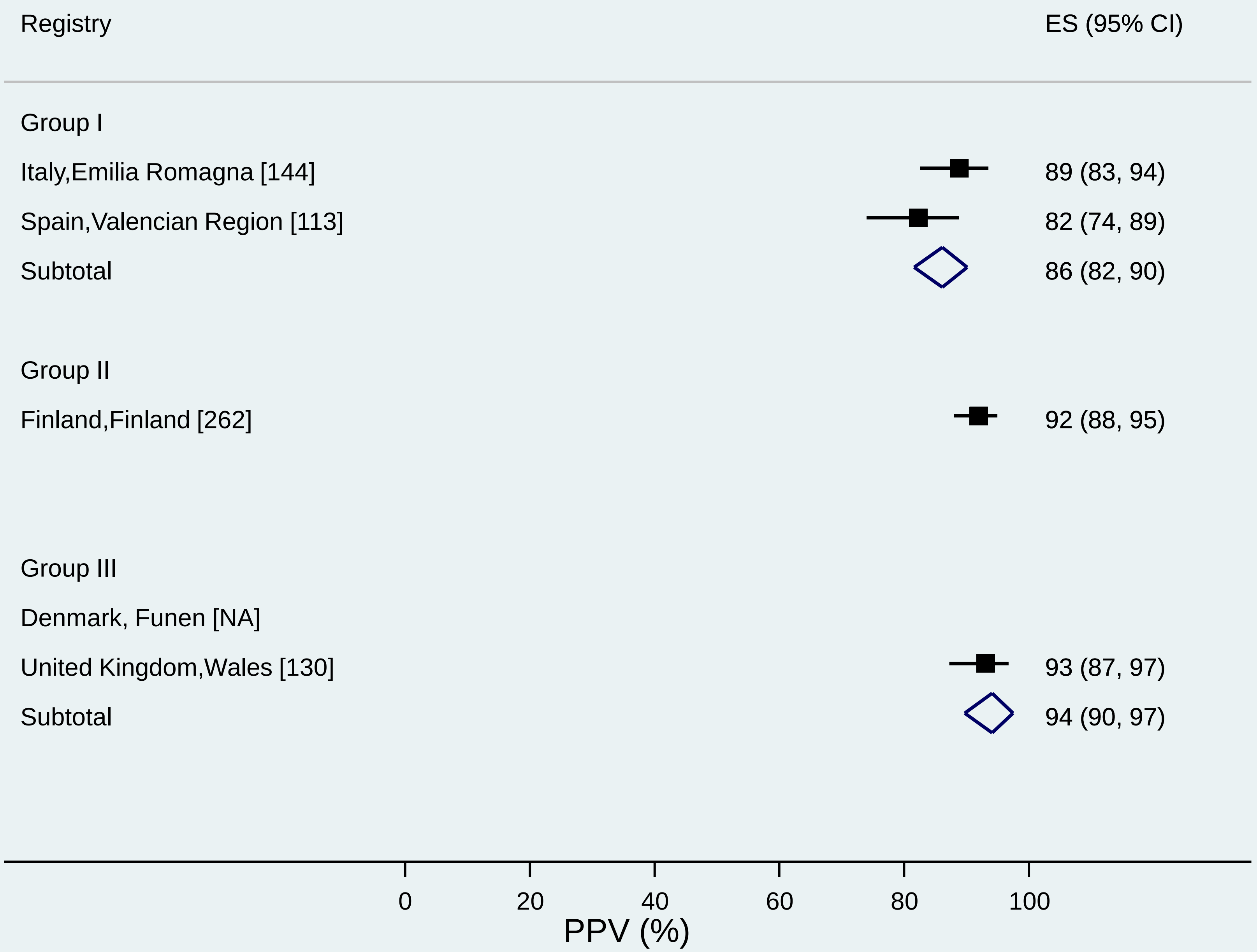

Anomalies detectable at birth

Estimates for PPV per registry, pooled estimates per group.

NA indicates that the number and/or the estimate cannot be reported because of release restrictions for small numbers.

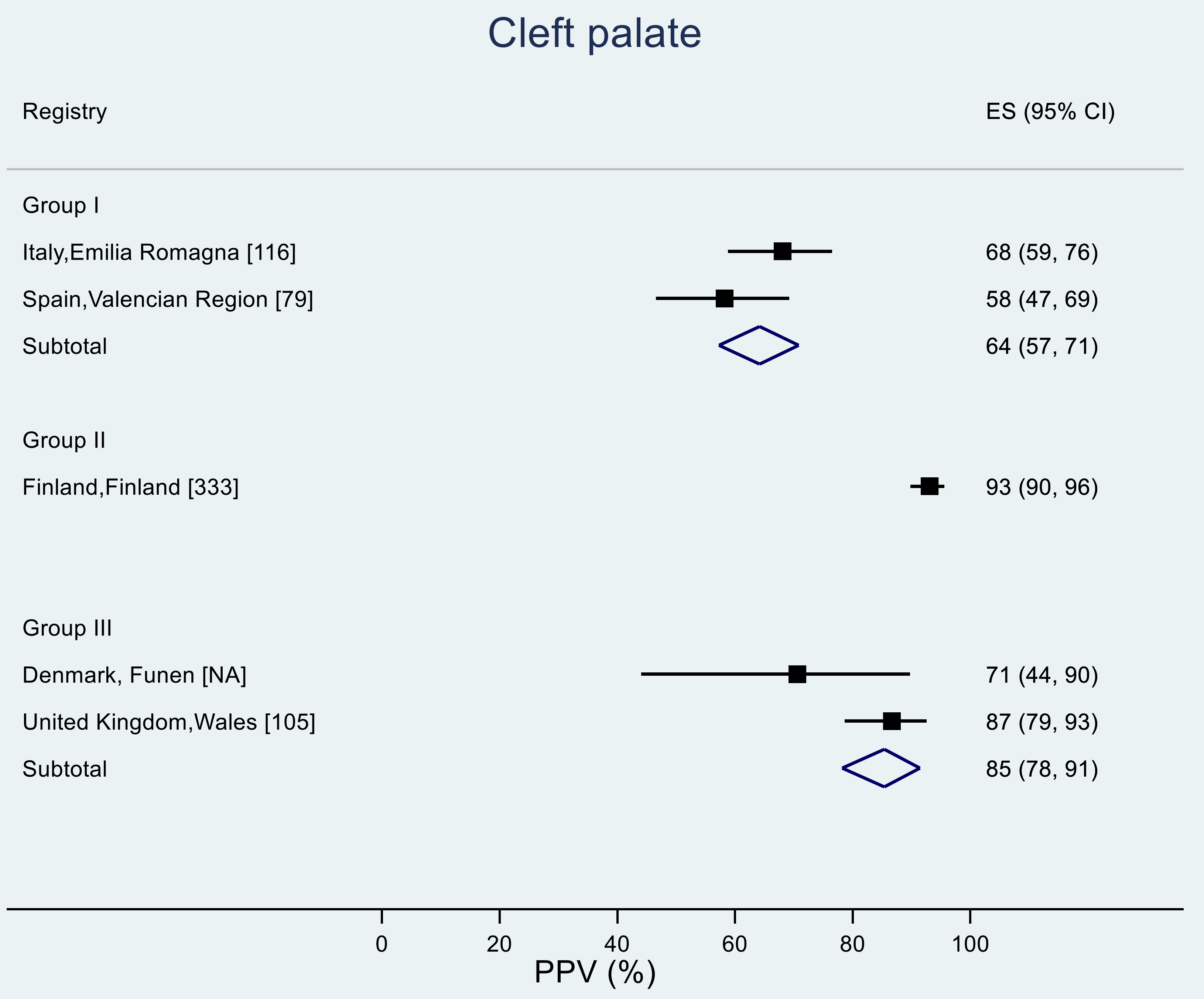

Anomalies detectable at birth

Estimates for PPV per registry, pooled estimates per group and overall pooled estimate.

NA indicates that the number and/or the estimate cannot be reported because of release restrictions for small numbers.

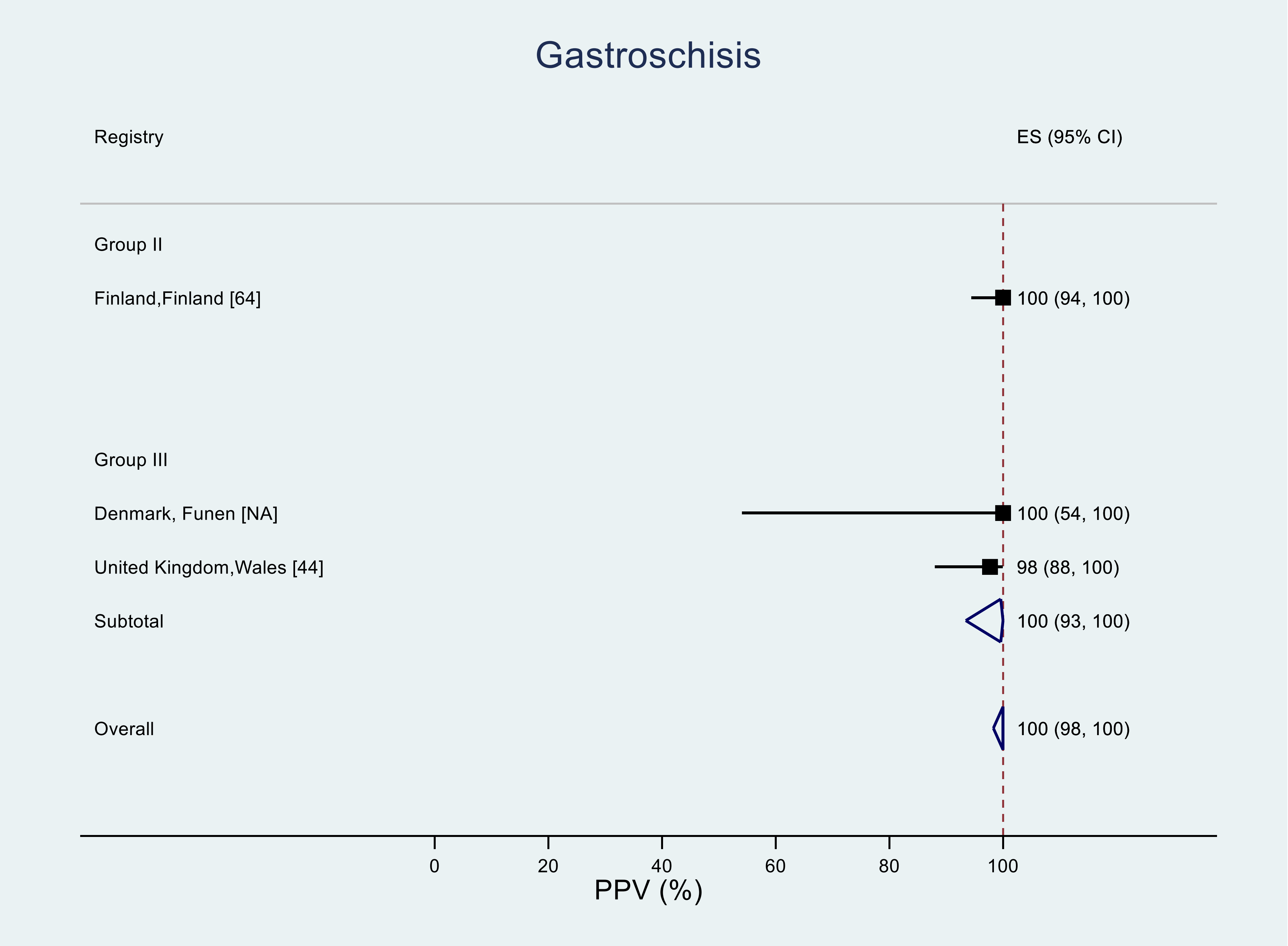

Anomalies detectable at birth

Estimates for PPV per registry, pooled estimates per group.

NA indicates that the number and/or the estimate cannot be reported because of release restrictions for small numbers.

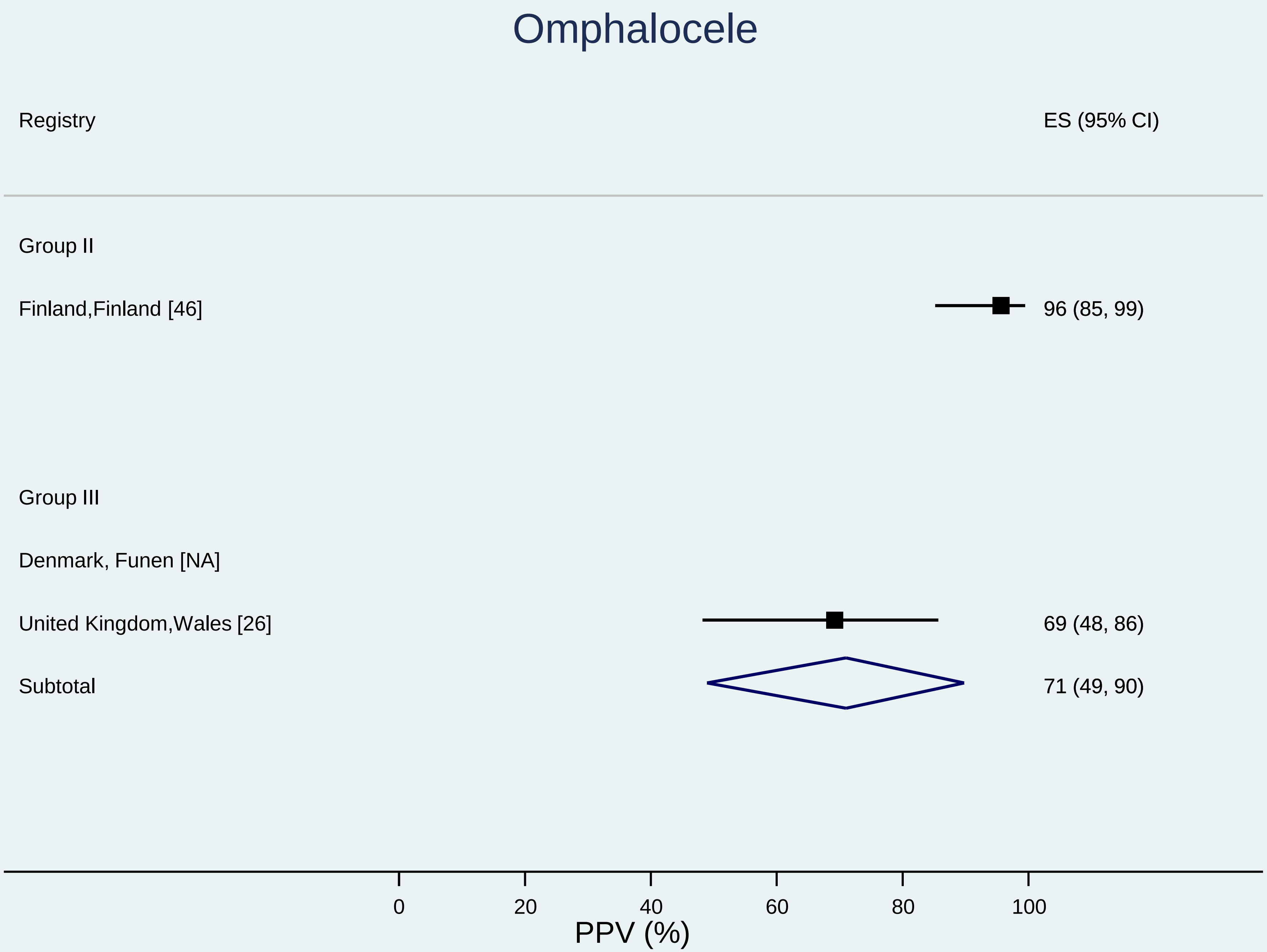

Anomalies detectable at birth

Estimates for PPV per registry, pooled estimates per group.

NA indicates that the number and/or the estimate cannot be reported because of release restrictions for small numbers.

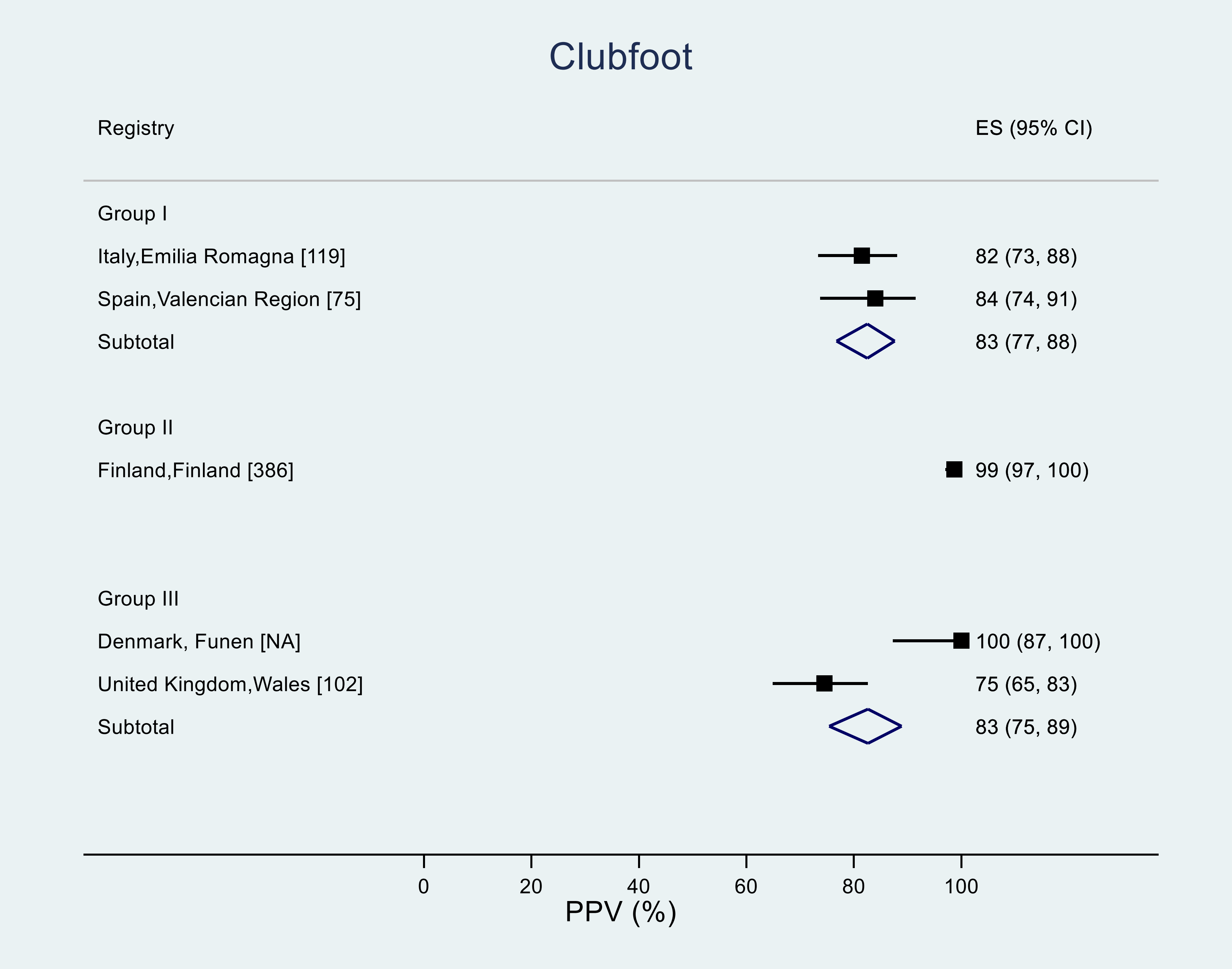

Anomalies with high prenatal detection rate

Estimates for PPV per registry, pooled estimates per group and overall pooled estimate.

NA indicates that the number and/or the estimate cannot be reported because of release restrictions for small numbers.

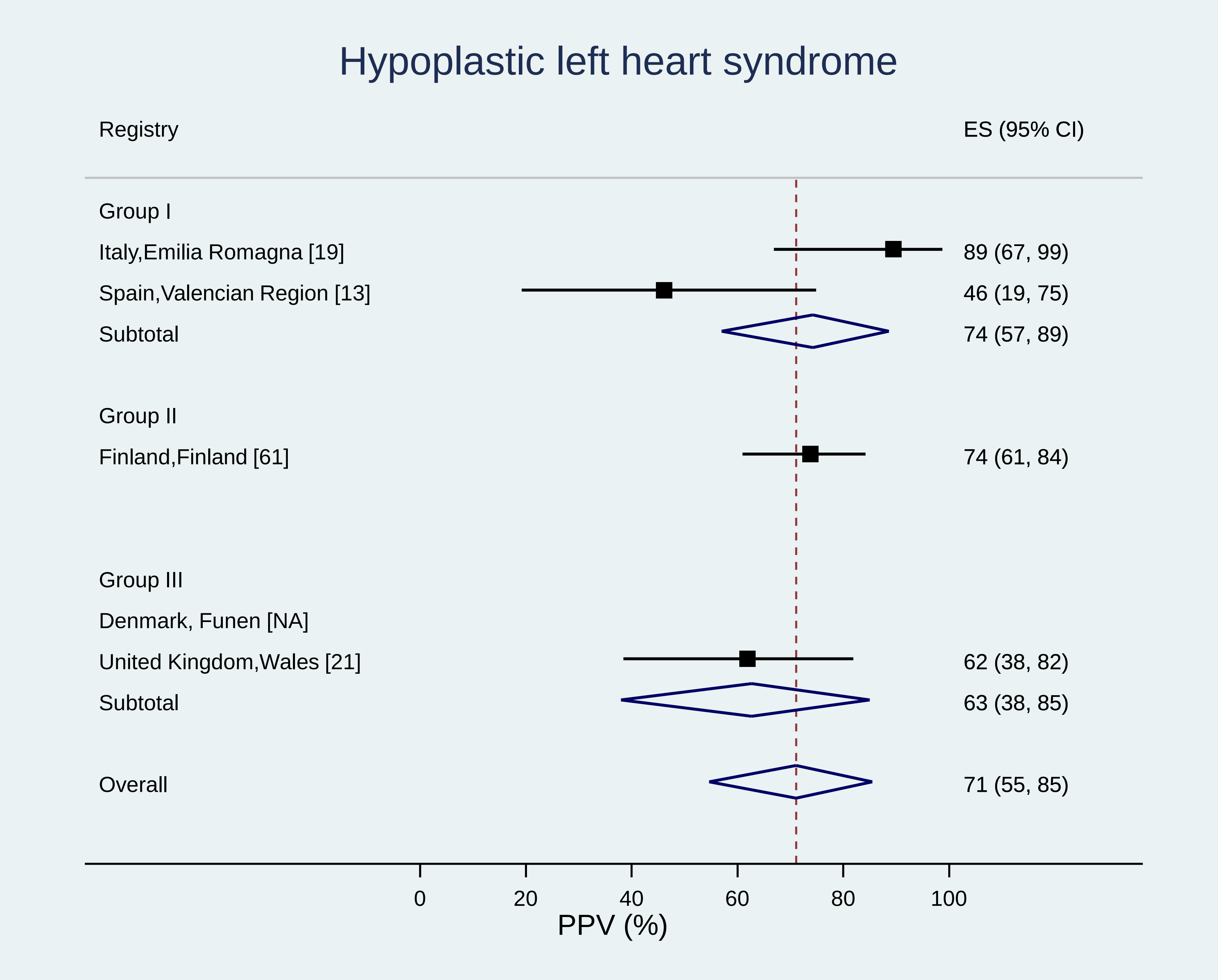

Anomalies with high prenatal detection rate

Estimates for PPV per registry, pooled estimates per group.

NA indicates that the number and/or the estimate cannot be reported because of release restrictions for small numbers.

Unilateral renal agenesis

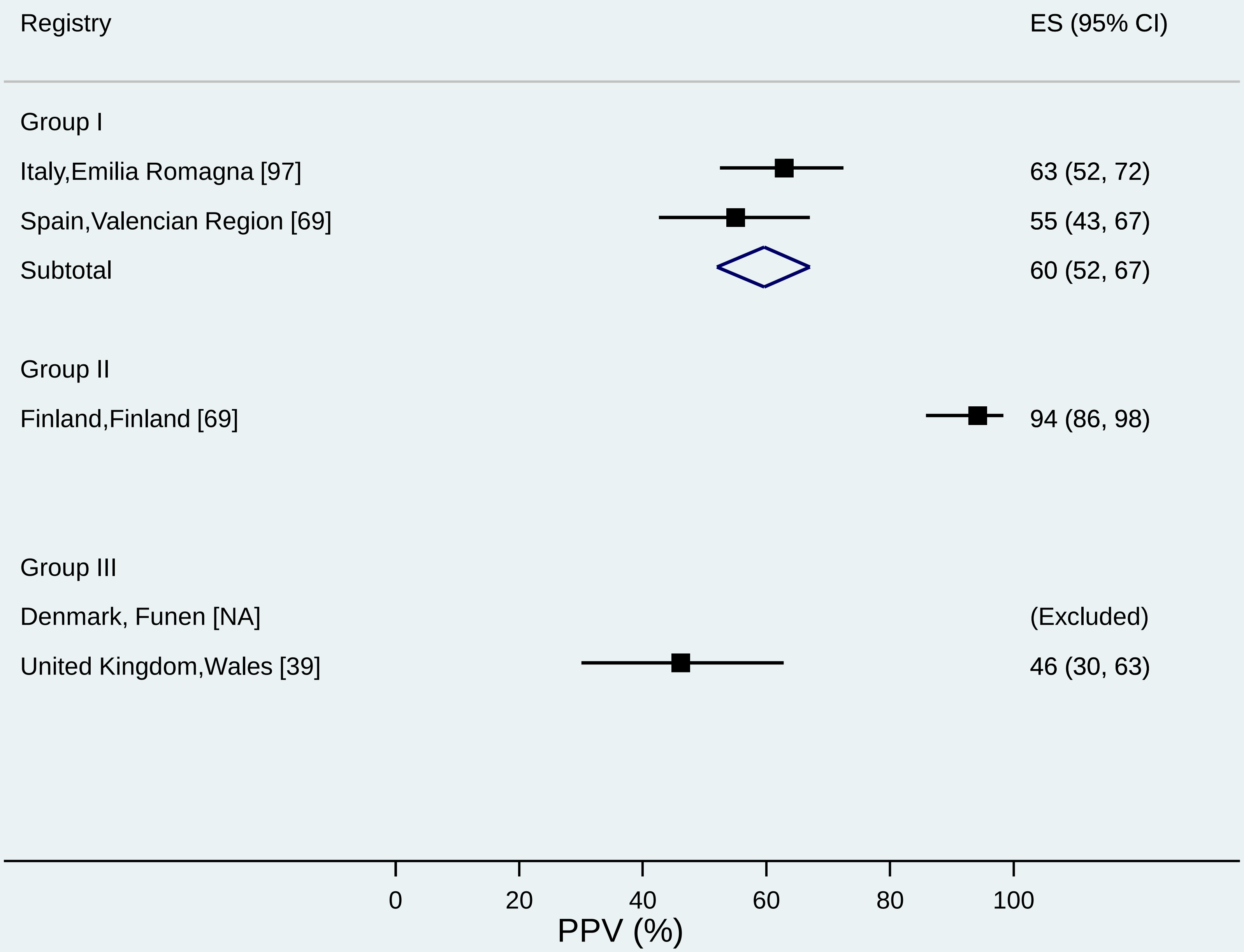

Anomalies with high prenatal detection rate

Estimates for PPV per registry, pooled estimates per group and overall pooled estimate.

NA indicates that the number and/or the estimate cannot be reported because of release restrictions for small numbers.

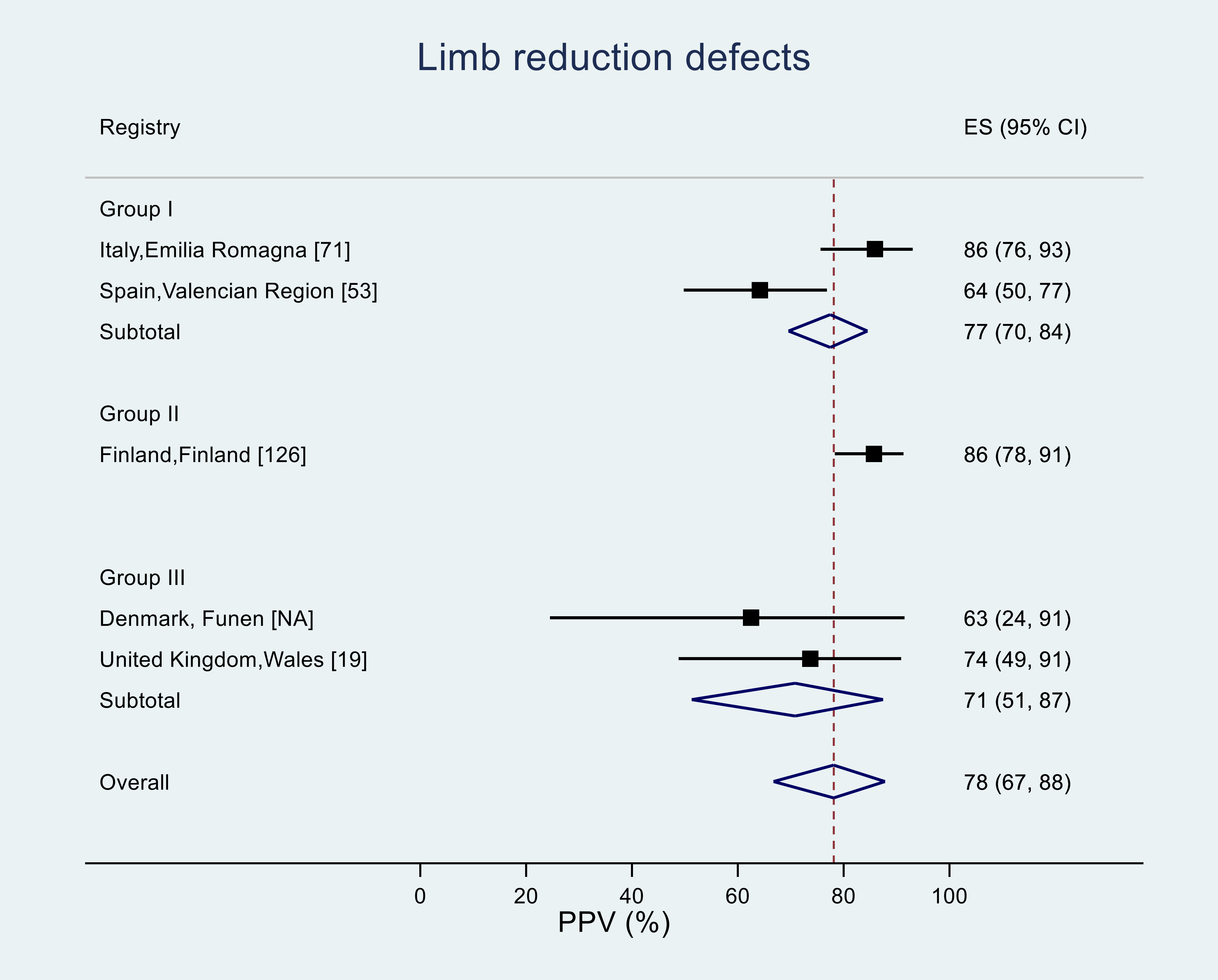

Anomalies usually diagnosed after discharge from the maternity unit

Estimates for PPV per registry, pooled estimates per group.

NA indicates that the number and/or the estimate cannot be reported because of release restrictions for small numbers.

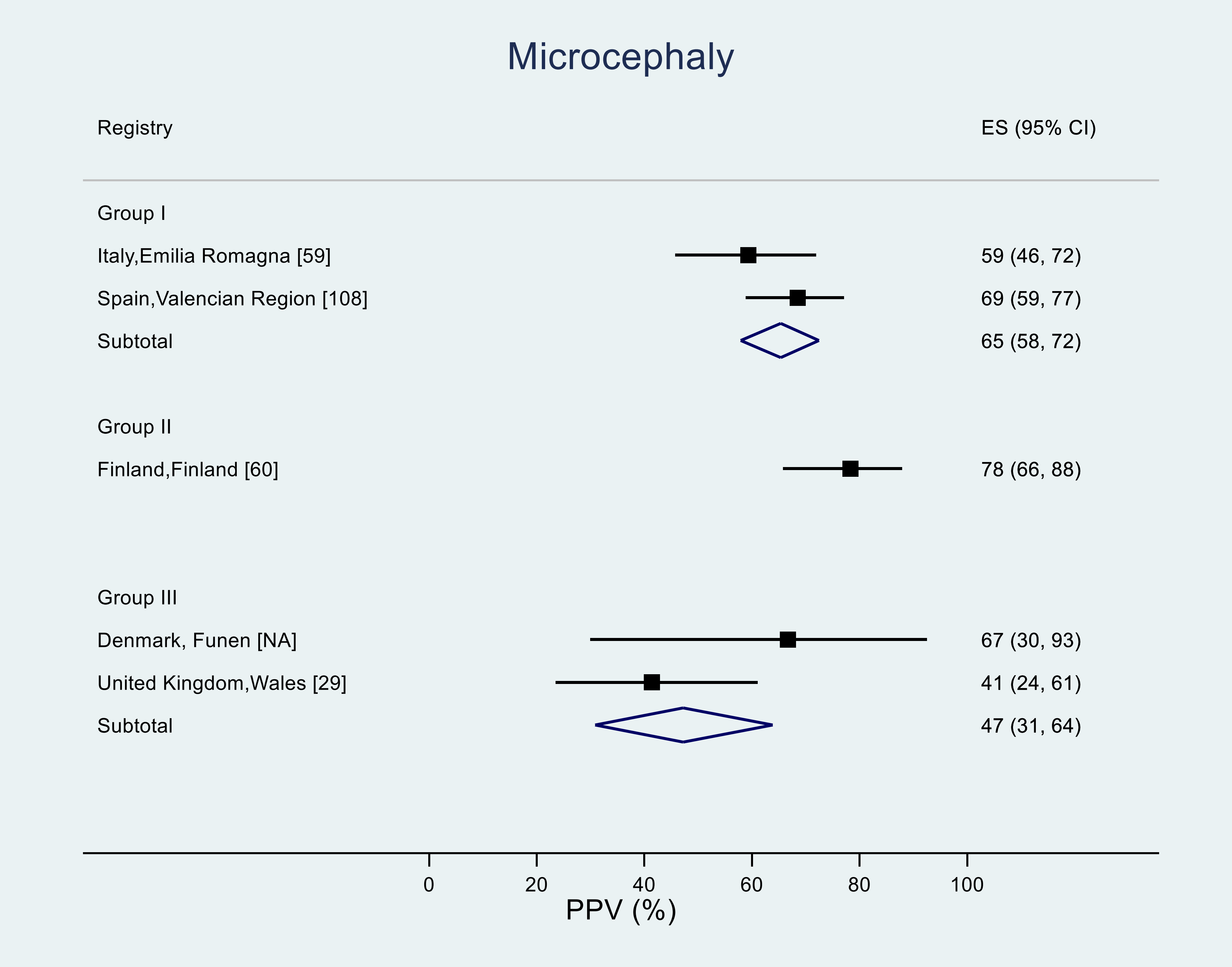

Anomalies usually diagnosed after discharge from the maternity unit

Estimates for PPV per registry, pooled estimates per group.

NA indicates that the number and/or the estimate cannot be reported because of release restrictions for small numbers.

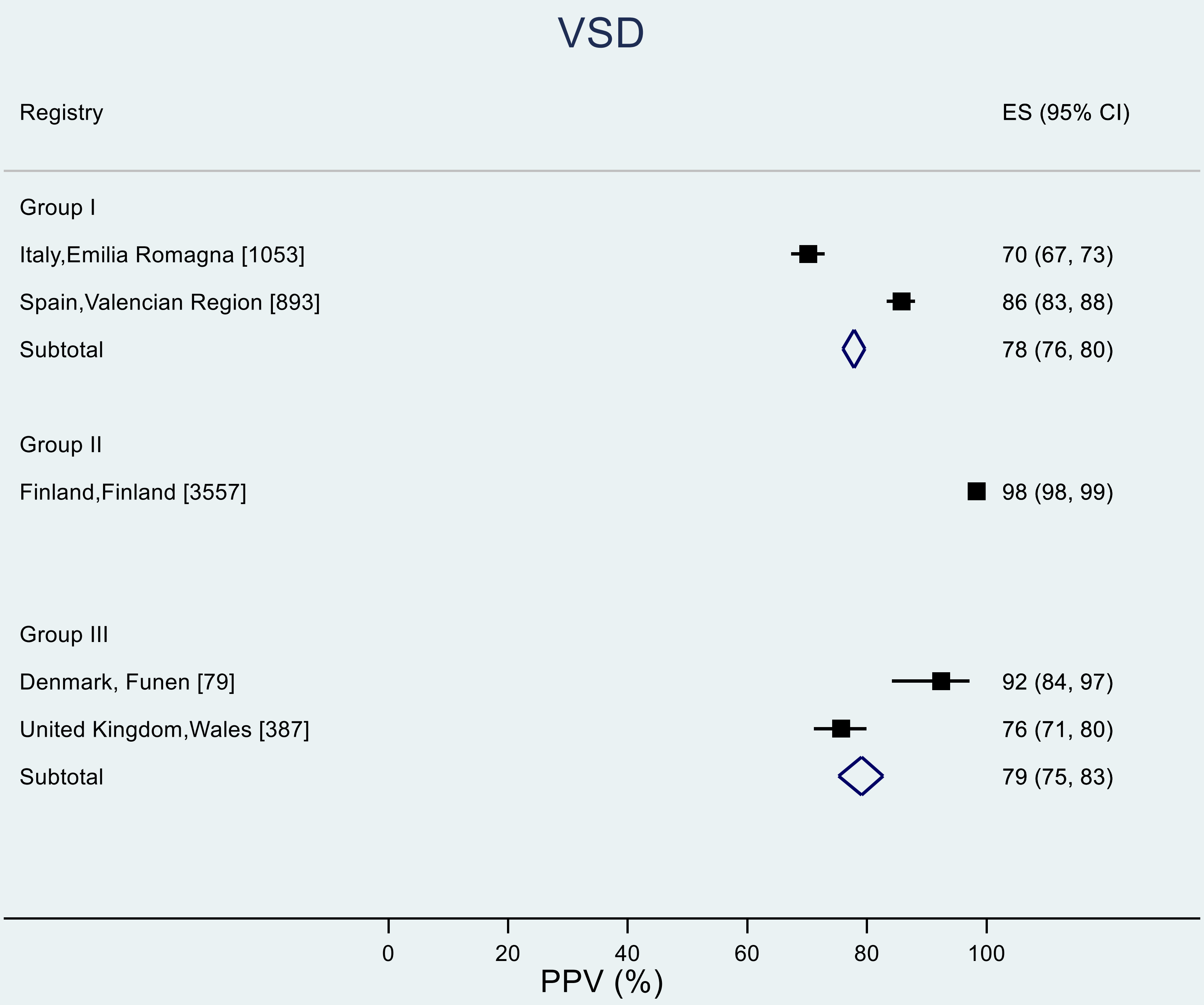

Anomalies usually diagnosed after discharge from the maternity unit

Estimates for PPV per registry, pooled estimates per group.

NA indicates that the number and/or the estimate cannot be reported because of release restrictions for small numbers.

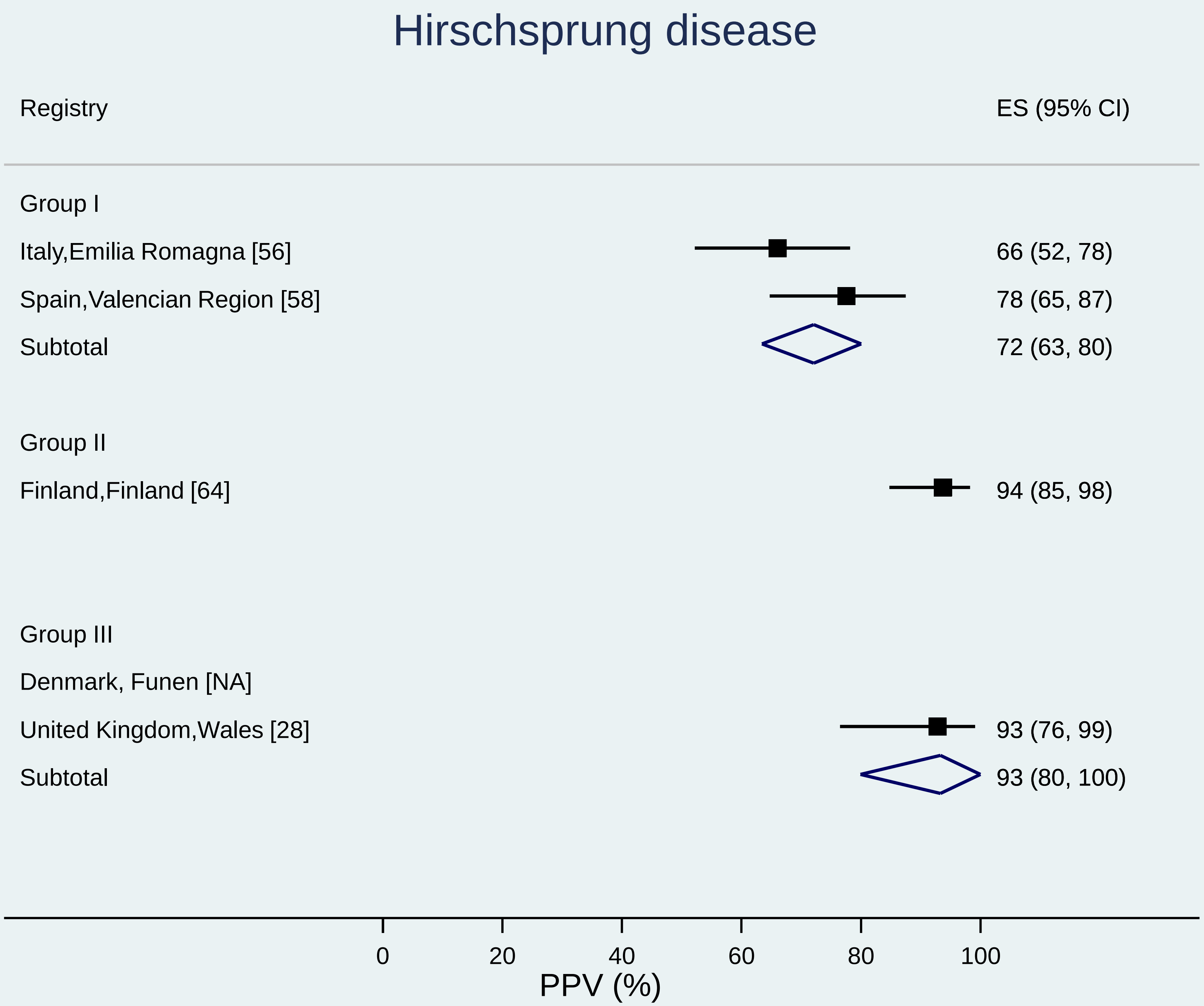

Anomalies that present in variable form between normal and abnormal

Estimates for PPV per registry, pooled estimates per group.

NA indicates that the number and/or the estimate cannot be reported because of release restrictions for small numbers.

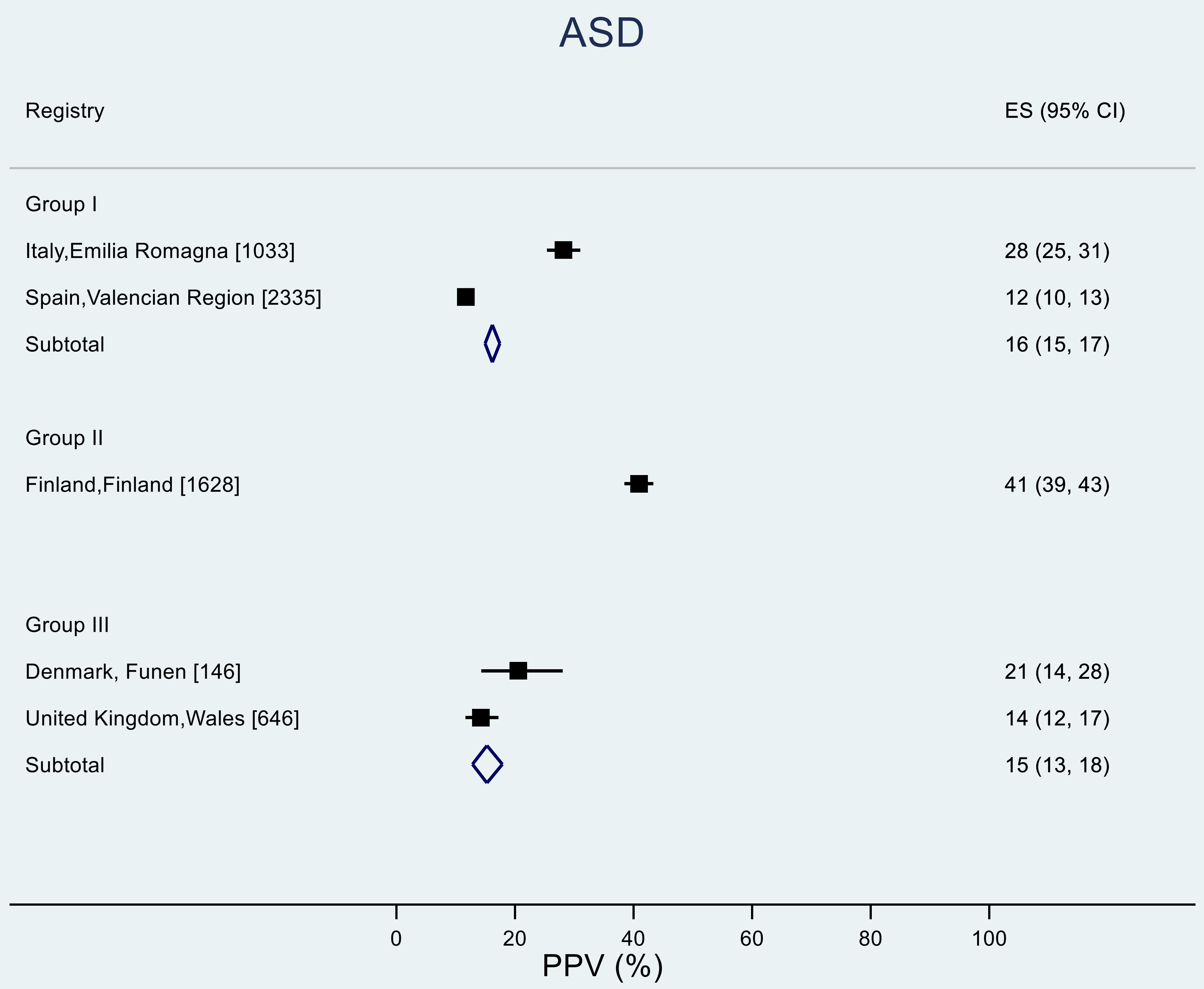

Anomalies that present in variable form between normal and abnormal

Estimates for PPV per registry, pooled estimates per group.

NA indicates that the number and/or the estimate cannot be reported because of release restrictions for small numbers.

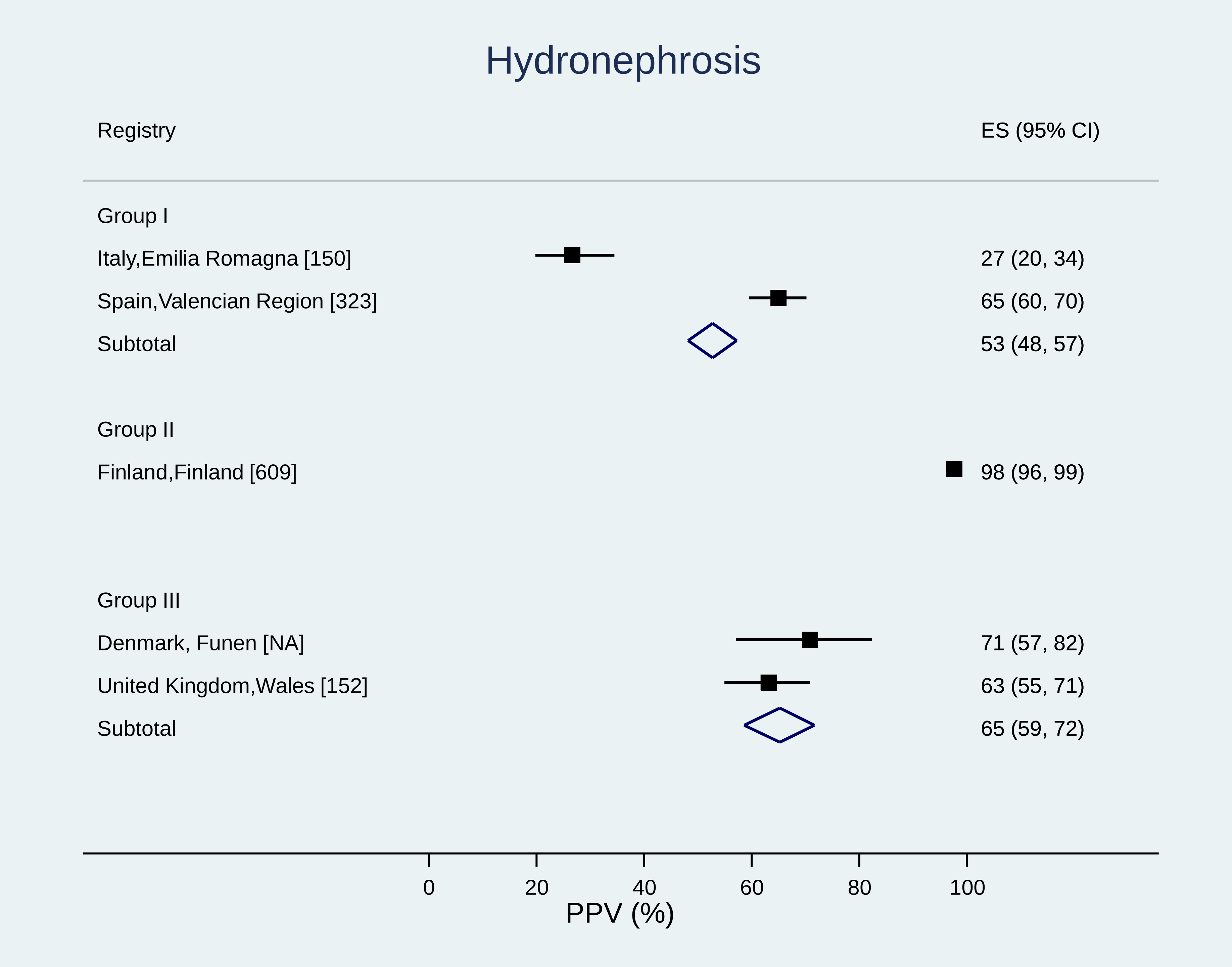

Anomalies that present in variable form between normal and abnormal

Estimates for PPV per registry, pooled estimates per group.

NA indicates that the number and/or the estimate cannot be reported because of release restrictions for small numbers.

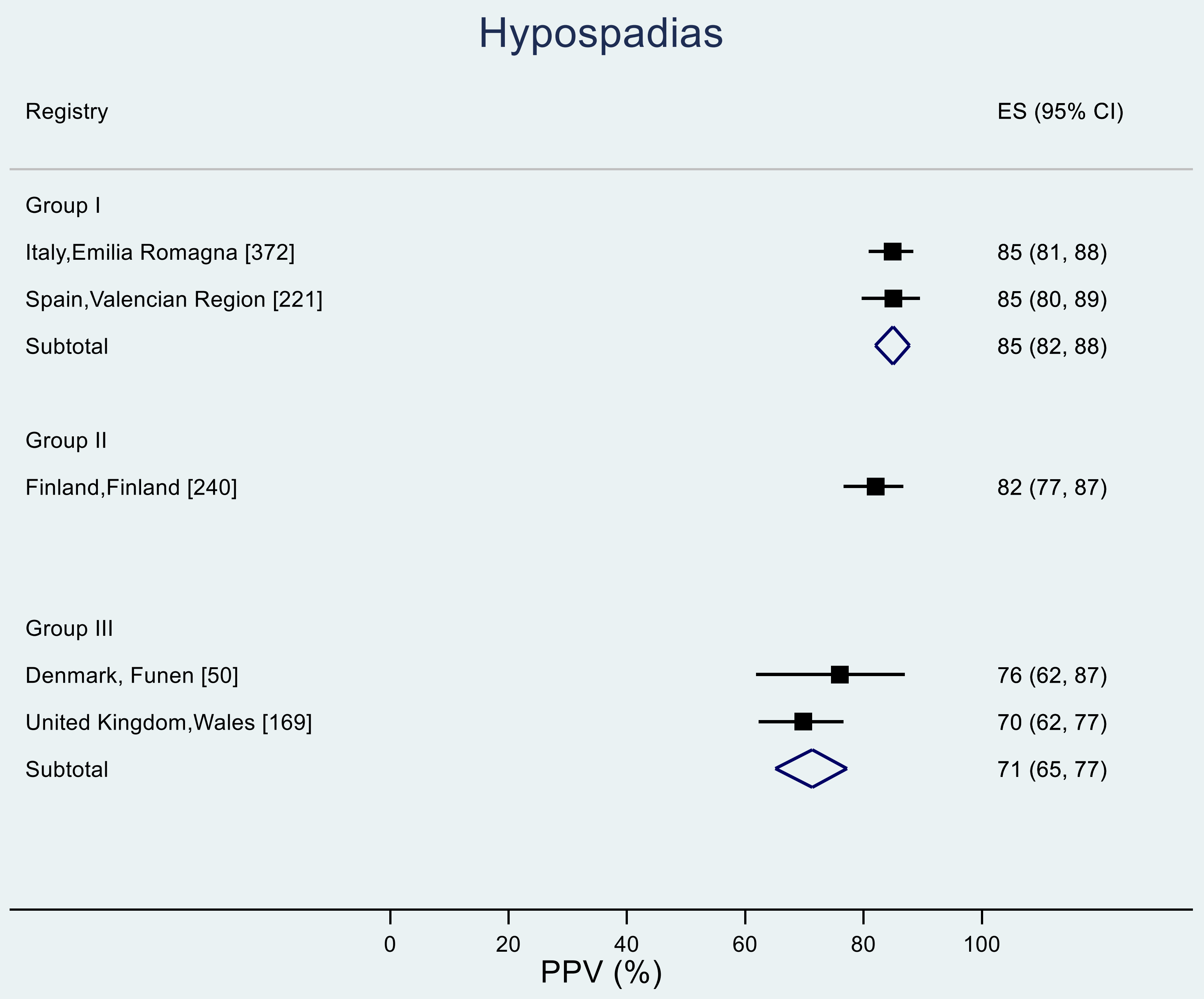

Chromosomal anomaly

Estimates for PPV per registry, pooled estimates per group.

NA indicates that the number and/or the estimate cannot be reported because of release restrictions for small numbers.

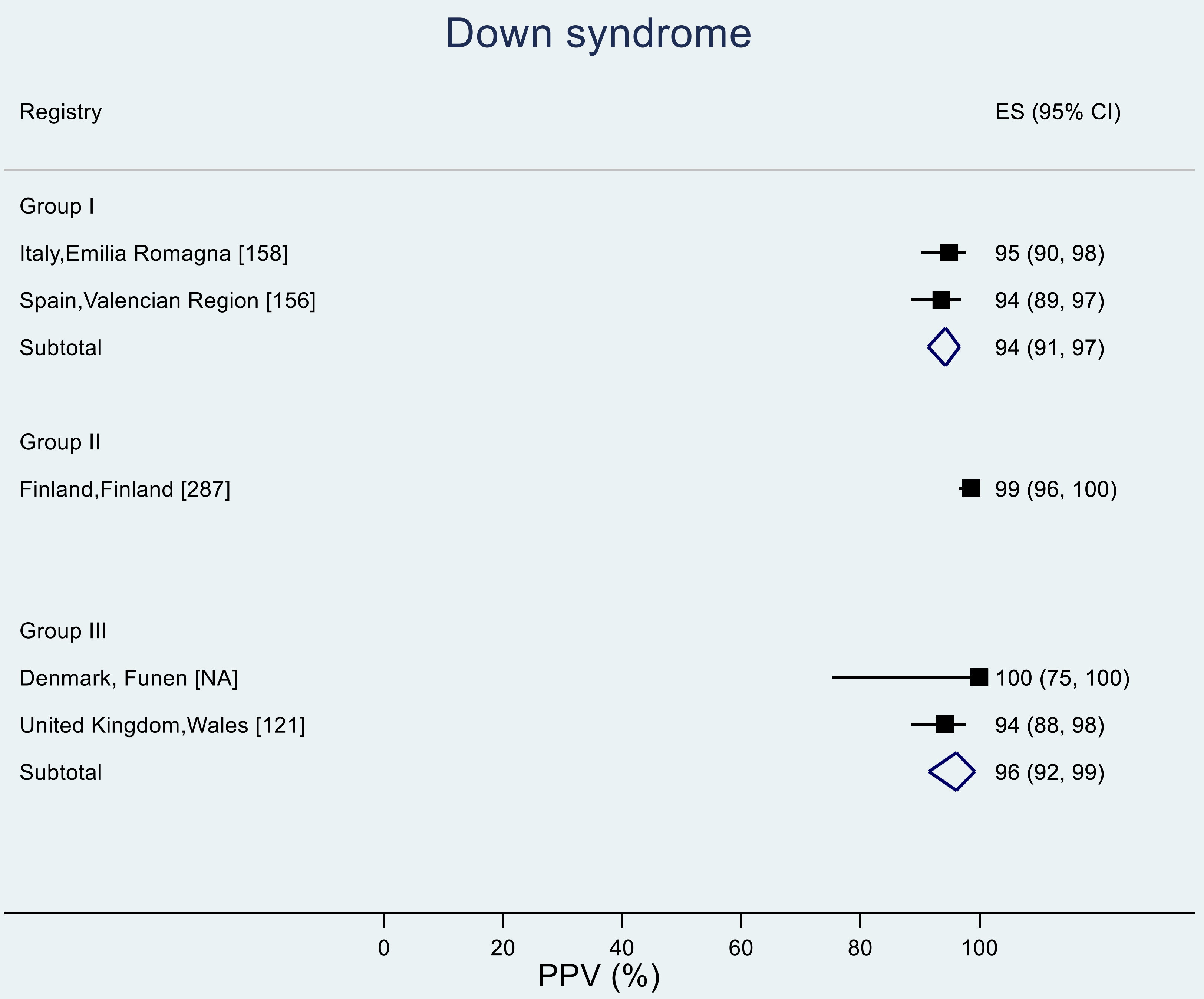

Mild anomaly

Estimates for PPV per registry, pooled estimates per group.

NA indicates that the number and/or the estimate cannot be reported because of release restrictions for small numbers.

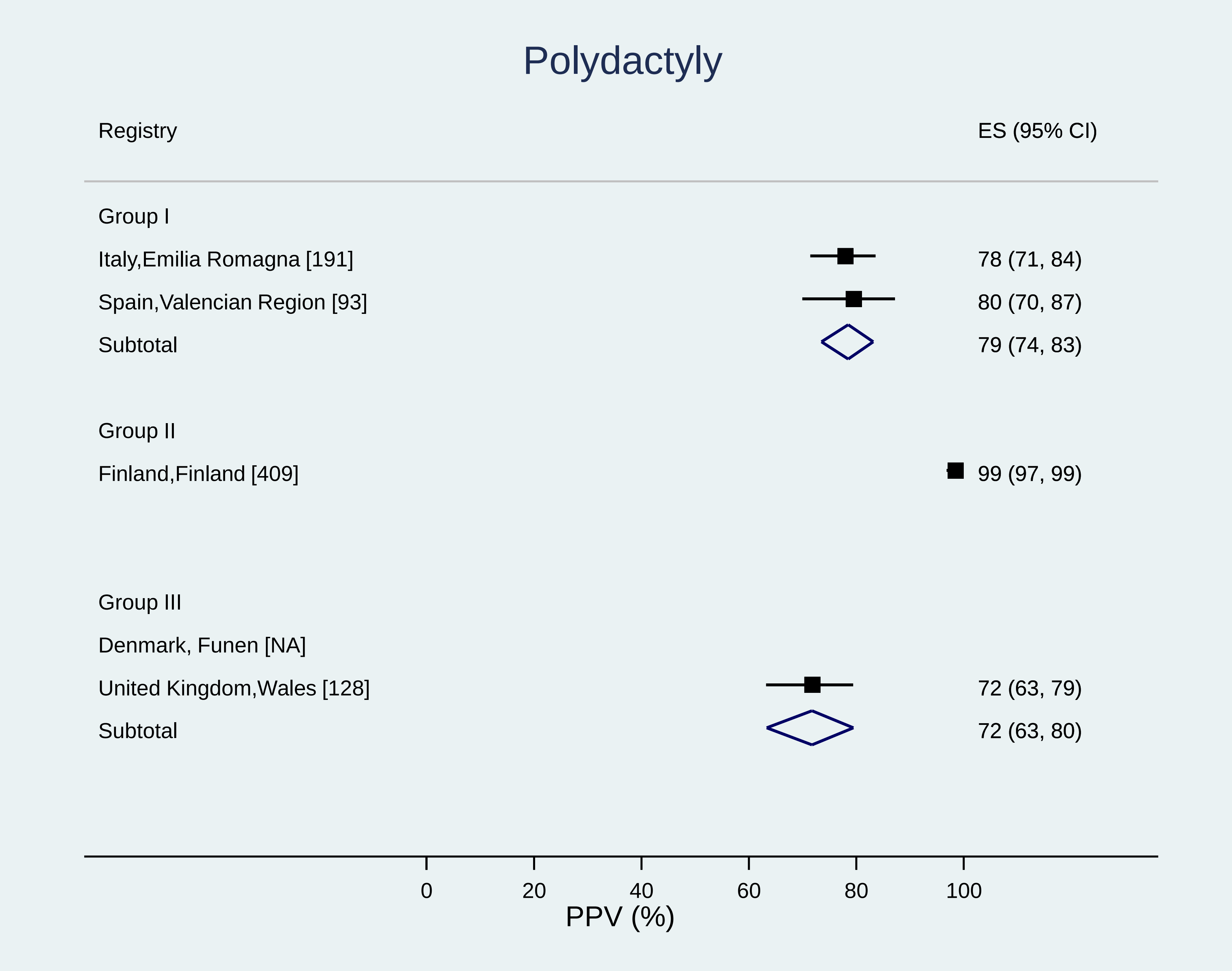

Supplement: Supplementary file 3 — Supplementary file3 (PDF 813 KB) [file 10654_2023_971_MOESM3_ESM.pdf]
